# Nuclear translocation of an aminoacyl-tRNA synthetase may mediate a chronic “integrated stress response”

**DOI:** 10.1016/j.celrep.2023.112632

**Published:** 2023-06-13

**Authors:** Julia A. Jones, Na Wei, Haissi Cui, Yi Shi, Guangsen Fu, Navin Rauniyar, Ryan Shapiro, Yosuke Morodomi, Nadine Berenst, Calin Dan Dumitru, Sachiko Kanaji, John R. Yates, Taisuke Kanaji, Xiang-Lei Yang

**Affiliations:** 1 Department of Molecular Medicine, Scripps Research Institute, La Jolla, CA 92037, USA; 2 Present address: R&D Laboratory for Innovative Biotherapeutics Science, Graduate School of Pharmaceutical Sciences, Kyushu University, Fukuoka, Japan; 3 These authors contributed equally; 4 Lead contact

## Abstract

Various stress conditions are signaled through phosphorylation of translation initiation factor eukaryotic initiation factor 2α (eIF2α) to inhibit global translation while selectively activating transcription factor ATF4 to aid cell survival and recovery. However, this integrated stress response is acute and cannot resolve lasting stress. Here, we report that tyrosyl-tRNA synthetase (TyrRS), a member of the aminoacyl-tRNA synthetase family that responds to diverse stress conditions through cytosol-nucleus translocation to activate stress-response genes, also inhibits global translation. However, it occurs at a later stage than eIF2α/ATF4 and mammalian target of rapamycin (mTOR) responses. Excluding TyrRS from the nucleus over-activates translation and increases apoptosis in cells under prolonged oxidative stress. Nuclear TyrRS transcriptionally represses translation genes by recruiting TRIM28 and/or NuRD complex. We propose that TyrRS, possibly along with other family members, can sense a variety of stress signals through intrinsic properties of this enzyme and strategically located nuclear localization signal and integrate them by nucleus translocation to effect protective responses against chronic stress.

## In brief

Jones and Wei et al. report that the nuclear translocation of tyrosyl-tRNA synthetase (TyrRS) under oxidative stress suppresses protein synthesis. This inhibition effect provides an additional cell survival mechanism during prolonged stress by transcriptional regulation, which functions distinctly from classic stress response pathways.

## Graphical Abstract

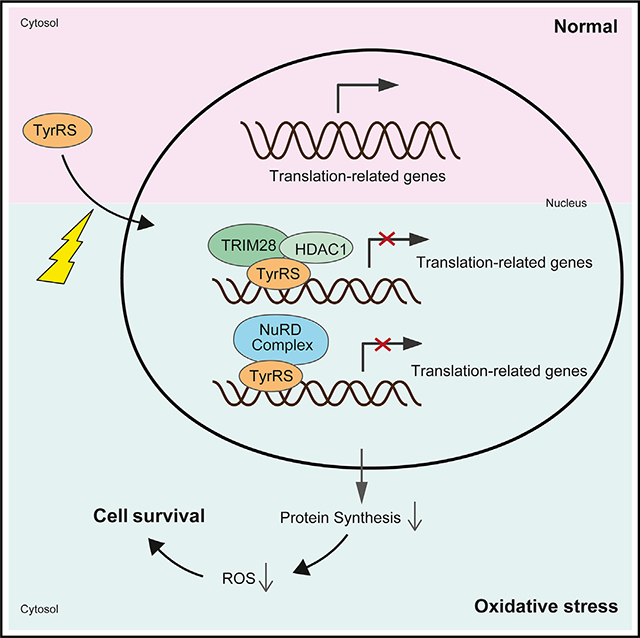

## INTRODUCTION

Regulation of mRNA translation is a key component of cellular stress response. Two major pathways—the regulation of mammalian target of rapamycin (mTOR) and the phosphorylation of initiation factor eukaryotic initiation factor 2α (eIF2α)—are well studied for translation control at the initiation step.^1–3^ Hypoxia and energy depletion disable eIF4E, via the regulation of mTOR, to prevent cap-dependent translation initiation.^4–7^ In response to a wide variety of stresses, extrinsic factors such as amino acid deprivation, and viral infection or intrinsic conditions such as endoplasmic reticulum (ER) stress caused by accumulation of unfolded proteins, cells activate a common adaptive pathway. This integrated stress response (ISR) reduces the availability of the translation initiation ternary complex (eIF2-GTP-Met-tRNA^Met^) via the phosphorylation of eIF2α by a family of upstream kinases.^8,9^ These two pathways are largely responsible for the acute reduction of translation in cells under stress conditions.

However, as a prolonged decrease in translation would be harmful, cells also subsequently trigger negative feedback loops that reactivate protein synthesis. Indeed, the ISR-induced phosphorylation of eIF2α inhibits global translation while selectively enabling the translation of the activating transcription factor 4 (ATF4) and CCAAT/enhancer-binding proteins homologous protein (CHOP). In addition to promoting the transcription of stress-response genes, ATF4 and CHOP also promote the expression of protein phosphatase 1 regulatory subunit 15A (PPP1R15A/GADD34), which dephosphorylates eIF2α to reinstate physiological protein synthesis.^10,11^ Moreover, many translation-related genes are direct targets of ATF4 and CHOP.^12^ In cells with unresolved stress, the ATF4/CHOP-mediated activation of translation causes ATP depletion and additional reactive oxygen species (ROS) production, leading to cell death.^12,13^ Thus, the eIF2α-ATF4/CHOP feedback loop prevents over-suppression of translation under stress but can also trigger cell death when the stress condition is not resolved quickly.

The largest translation-related gene family targeted by ATF4 and CHOP is aminoacyl-tRNA synthetases (aaRSs).^12,14^ These cytoplasmic enzymes catalyze an ATP-dependent tRNA aminoacylation reaction, which provides building blocks for ribosomal synthesis of growing peptides according to the genetic code. Interestingly, many aaRSs are also found in the nucleus of eukaryotic cells, where translation generally does not occur. The initial hypothesis was that aaRSs function here in proofreading newly synthesized tRNAs.^15,16^ However, later findings suggest that the nucleus-localized aaRSs are involved in regulating a wide range of biological processes including vascular development, inflammation, energy production, and stress responses, mainly due to their distinctive abilities to interact with the transcriptional machinery and DNA.^17–20^

Interestingly, we found that the nuclear localization signal (NLS) sequence in human tyrosyl-tRNA synthetase (TyrRS; gene name YARS) is directly involved in tRNA binding at the anticodon.^21^ As a result, the nuclear translocation of the synthetase is negatively regulated by its cognate tRNA in the cytosol.^21^ Consistently, angiogenin, a stress-activated ribonuclease that cleaves tRNA at the anticodon loop,^22,23^ stimulates the nuclear translocation of TyrRS.^19^ In fact, TyrRS nuclear translocation can be stimulated by a wide variety of stress conditions, including oxidative stress, ER stress, heat shock, and serum starvation.^19,24^ The nucleus-localized TyrRS strongly protects against UV-induced DNA double-strand breaks in zebrafish and does so by upregulating DNA damage repair genes, such as BRCA1 and RAD51, through the ATF E2F1.^19^ Most recently, nuclear TyrRS-mediated ATR lysine tyrosylation was also shown to activate early-stage DNA damage responses and protect against heart failure.^25^ Thus, TyrRS has been established as a stress-response protein through its nuclear functions.

Here, we set out to investigate whether TyrRS also regulates translation through its stress-induced cytosol-nucleus translocation. Indeed, we found that nuclear TyrRS inhibits global translation and that the effect of nuclear TyrRS in translation control happens after the initiation of the mTOR and eIF2α responses and thus provides the cells with a second chance to adapt and survive through the stress conditions. Apart from this difference in timing, the ability of TyrRS to respond to a wide variety of stress factors and to effect translation inhibition while selectively activating stress-response genes resembles the function of ISR. Therefore, TyrRS may be considered a core effector for an ISR-like response to aid cells during prolonged stress. This response may also involve other tRNA synthetase family members to collectively sense a larger variety of stress factors and instigate diverse, protective responses to restore cellular homeostasis through their cytosol-nucleus translocation.

## RESULTS

### Nuclear TyrRS inhibits global translation during late-stage oxidative stress

**Figure 1.**
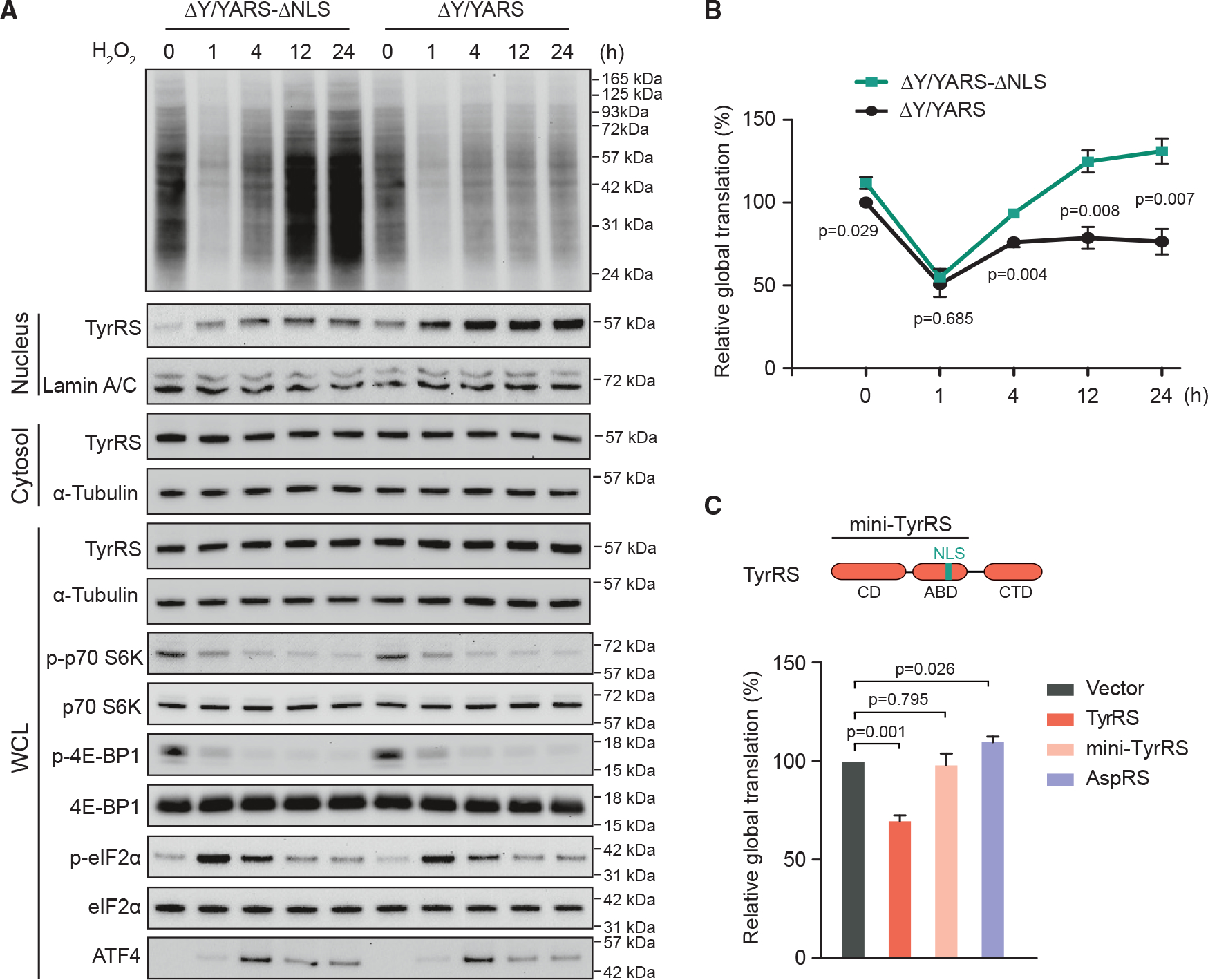
Nuclear TyrRS inhibits global mRNA translation during late-stage oxidative stress (A) SUnSET analysis of nuclear TyrRS-deficient or control HEK293 cells. Cells were treated with H_2_O_2_ for the indicated time. TyrRS nuclear localization was analyzed by cell fractionation followed by western blot. Total protein expression was examined using whole-cell lysate (WCL). (B) SUnSET western blot quantification with the intensities normalized to α-tubulin for each treatment and the ΔY/YARS time point 0 set to 100%. n = 3, biological replicates, Student’s t test. (C) Domain architecture of TyrRS and mini-TyrRS. CD, catalytic domain; ABD, anti-codon binding domain; CTD, C-terminal EMAP II-like domain; NLS, nuclear localization sequence. Quantified SUnSET analysis to show translation inhibition by overexpression of wild-type TyrRS but not mini-TyrRS or AspRS (aspartyl-tRNA synthetase). n = 3, biological replicates, Student’s t test. See Figure S2 for transgene expression levels in HEK293 cells.

Oxidative stress can activate eIF2α through all known upstream kinases^8,26,27^ and can inhibit mTOR signaling^6,7^ in mammalian cells. To evaluate the effect of TyrRS nuclear entry on global translation under oxidative stress, we established stable HEK293 cells expressing endogenous levels of wild-type TyrRS (ΔY/YARS) or NLS-mutated TyrRS (ΔY/YARS-ΔNLS)^19^ (Figure S1). The ΔNLS mutation (^242^KKKLKK^247^ to NNKLNK) specifically excludes TyrRS from the nucleus (Figure S1) without affecting its enzymatic activity required for translation in the cytosol.^21^ TyrRS nuclear localization is stimulated in ΔY/YARS cells under oxidative stress but is deficient in ΔY/YARS-ΔNLS cells with or without stress (Figure S1). H_2_O_2_ treatment, which causes oxidative stress, affects global translation in a time-dependent manner (Figures 1A and 1B). Shortly after H_2_O_2_ treatment (1 h), a sharp decrease (~50%) in global translation was observed using the SUnSET technique^28^ in both ΔY/YARS (“normal”) and ΔY/YARS-ΔNLS (nuclear TyrRS-deficient) cells. After a longer period of H_2_O_2_ treatment (4, 12, or 24 h), the global translation level in “normal” cells recovered to about 70%; in comparison, nuclear TyrRS-deficient cells exhibit faster recovery of translation and reached a higher level of translation after 4 h H_2_O_2_ treatment (Figures 1A and 1B). These results suggest that nuclear TyrRS functions to inhibit and prevent over-stimulation of global translation when cells are under prolonged oxidative stress.

### The mTOR and eIF2α pathways do not contribute to the inhibitory effect of nuclear TyrRS

The sharp decrease in global translation after H_2_O_2_ treatment (1 h) is concurrent with both ISR activation and mTOR inhibition, which are indicated by the phosphorylation of Ser^29^ in eIF2α and the subsequent expression of ATF4 and by the decrease in the levels of phosphorylated eIF4E-binding protein 1 (4E-BP1) and p70 ribosomal protein S6 kinase (S6K),^1^ respectively (Figure 1A), suggesting that ISR- and mTOR-mediated controls of translation initiation are responsible for the initial decrease in global translation observed here. Importantly, there is no obvious difference in the phosphorylation of eIF2α, 4E-BP1, and S6K between ΔY/YARS and ΔY/YARS-ΔNLS cells, suggesting that nuclear TyrRS inhibits translation through an ISR- and mTOR-in-dependent mechanism. Moreover, the effect of nuclear TyrRS on translation is manifested later, suggesting a slower, possibly transcriptional mechanism.

### Translation inhibitory effect of TyrRS is independent of its cytosolic enzymatic activity

To exclude the possibility that oxidative stress-induced nuclear translocation of TyrRS inhibits protein synthesis simply by depleting the cytosolic pool of the synthetase, we overexpressed V5-tagged wild-type TyrRS in HEK293 cells (Figure S2). Interestingly, overexpression of TyrRS did not promote but rather suppressed global translation (Figure 1C), presumably through increased nuclear translocation of TyrRS.^19,21^ We also tested mini-TyrRS, the evolutionarily conserved catalytic core of TyrRS constituting the catalytic domain (CD) and the anticodon-binding domain (ABD) where the NLS is located^21^ (Figure 1C). However, mini-TyrRS lacks the C-terminal EMAP II-like domain (CTD), to which many of the non-canonical functions of TyrRS are inherent and which might aid nucleic acid binding.^30,31^ Overexpression of mini-TyrRS, which is fully active for tRNA aminoacylation,^31^ did not affect global translation (Figure 1C), further confirming that the translation inhibitory effect of TyrRS is independent of its enzymatic activity.

### Nuclear TyrRS is protective against cell death induced by oxidative stress

**Figure 2.**
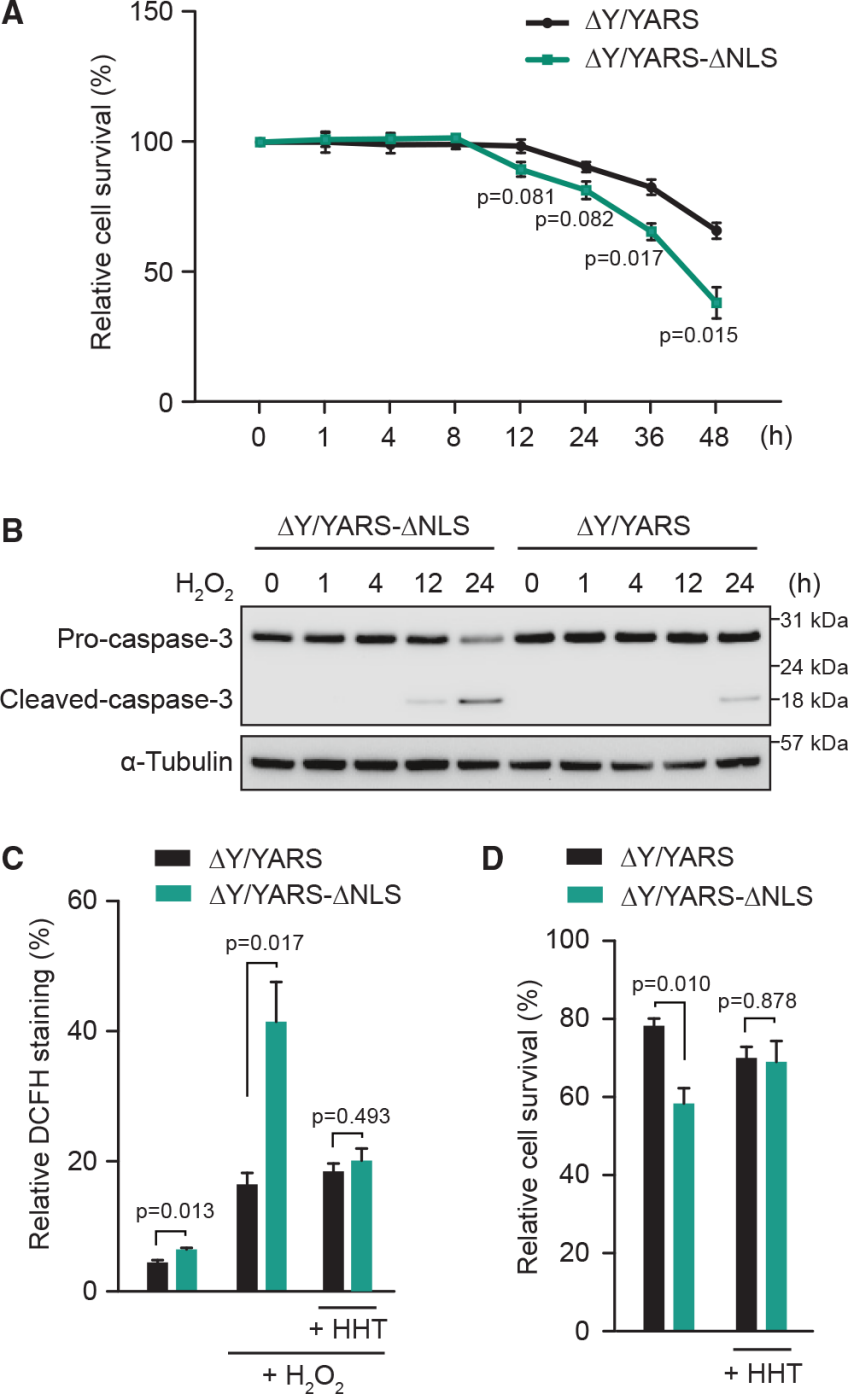
Nuclear TyrRS protects against cell death induced by oxidative stress (A) TyrRS nuclear exclusion enhances cell death caused by H_2_O_2_ treatment. Cell survival of ΔY/YARS at time point 0 set to 100%. n = 3, biological replicates, Student’s t test. (B) Nuclear TyrRS-deficient cells have enhanced apoptosis as detected bycaspase-3 cleavage western blot. (C) Nuclear TyrRS prevents ROS over-accumulation under H_2_O_2_ treatment for 36 h, but the effect is ablated by HHT treatment. ROS accumulation was detected by CM-H_2_DCFDA (DCFH). n = 3, biological replicates, Student’s t test. (D) Inhibition of translation by HHT reduces cell death in nuclear TyrRS-deficient cells. Cells treated 24 h with or without HHT followed by 36 h H_2_O_2_ treatment. Relative cell viabilities normalized to ΔY/YARS without treatment. n = 3, biological replicates, Student’s t test.

Dysregulation of translation often leads to cell death.^32–34^ After a 12 h H_2_O_2_ treatment, the nuclear TyrRS-deficient (ΔY/YARS-ΔNLS) cells exhibited more cell death than cells with compensatory expression of wild-type TyrRS (ΔY/YARS) (Figure 2A). The reduced viability of ΔY/YARS-ΔNLS cells was accompanied by increased levels of cleaved caspase 3 (Figures 2B and S3), indicating elevated apoptosis in nuclear TyrRS-deficient cells. Thus, nuclear TyrRS is protective against cell death induced by oxidative stress. Importantly, this effect was at least partially independent of the protective role of nuclear TyrRS against DNA damage mediated by transcriptional activation of E2F1,^19^ as knockdown of E2F1 did not diminish the cell-protective effect of nuclear TyrRS against oxidative stress (Figures S4A and S4B).

### Cell protection by nuclear TyrRS is predominately mediated by its impact on translation

Stress-induced cell death has been linked to overactivation of translation and subsequent intracellular ROS accumulation.^12,13^ Without H_2_O_2_ treatment, the basal level of ROS in nuclear TyrRS-deficient cells was slightly higher than in control cells (Figure 2C), correlating with increased basal levels of translation in these cells (Figures 1A and 1B). After H_2_O_2_ treatment, the difference in ROS level in ΔY/YARS and ΔY/YARS-ΔNLS cells was dramatically increased (Figure 2C). However, this difference was abolished if we pre-treated the cells with the translation elongation inhibitor homoharringtonine (HHT) (Figure 2C), suggesting that the ROS increase in nuclear TyrRS-deficient cells was translation dependent. We ruled out the possibility that nuclear TyrRS reduced ROS accumulation by promoting a common antioxidative stress response. No obvious difference in the levels of antioxidative stress-response proteins, such as SOD1, catalase, and Ucp2, was observed in ΔY/YARS versus ΔY/YARS-ΔNLS cells (Figure S5). Interestingly, HHT treatment also diminished the viability difference between nuclear TyrRS-deficient cells and “normal” cells (Figure 2D), suggesting that the protective effect of nuclear TyrRS against cell death is predominantly mediated by its impact on translation.

### Translation inhibition effect of nuclear TyrRS confirmed in mouse fibroblasts

To confirm that nuclear TyrRS inhibits global translation in another mammalian cell system, we used mouse fibroblasts prepared from nuclear TyrRS-deficient mice (Yars^ΔNLS/ΔNLS^). In agreement with our findings in the human cells, the ΔNLS mutations disrupted nuclear import of TyrRS in the mouse cells (Figure S6A) and led to overactivation of translation after a 4 h H_2_O_2_ treatment (Figures S6B and S6C). Moreover, compared with *Yars*^+/+^ cells, the *Yars*^ΔNLS/ΔNLS^ fibroblasts showed a reduced viability in response to oxidative stress (Figures S6D and S6E), which was accompanied by an increase in ROS production (Figure S6F). Again, the application of HHT, as well as another translation elongation inhibitor, cycloheximide (CHX), reduced the ROS production in *Yars*^ΔNLS/ΔNLS^ cells and eliminated the impact of TyrRS nuclear deficiency on ROS production and cell viability (Figures S6E and S6F).

Interestingly, in eIF2α phosphorylation-deficient (eIF2α^S51A^) mouse fibroblasts,^35^ we persistently observed overactivation of translation after 4 h oxidative stress when cells are deficient of nuclear TyrRS (Figure S7), confirming that the impact of nuclear TyrRS is independent of ISR. Notably, in the ISR-deficient background, translation inhibition still happens immediately after the oxidative stress, suggesting that ISR-independent mechanisms are responsible for the translation inhibition in ISR-deficiency cells.^36–38^ These ISR-independent mechanisms may include mTOR inhibition as we have observed in H_2_O_2_-treated HEK293 cells (Figure 1A). Because the inhibition on mTOR under the oxidative stress was sustained for at least 24 h (Figure 1A), it could also contribute to translation control during chronic stress. However, in mouse fibroblasts, mTOR remains active throughout 24 h H_2_O_2_ treatment (Figure S8A), indicating that mTOR inhibition is not required for oxidative stress-induced translation control in these cells.

To further probe the role of mTOR in translation control in mouse fibroblasts, we treated the cells with H_2_O_2_ as well as an mTOR inhibitor, Torin, which suppressed S6K phosphorylation for at least 12 h (Figure S8B). As expected, the effect of nuclear TyrRS on translation is prominent after a 12 h H_2_O_2_ treatment. Interestingly, the addition of Torin had a large impact on global translation in *Yars*^ΔNLS/ΔNLS^ cells but not in *Yars*^+/+^ cells at this time point (Figure S8C), suggesting that in the absence of nuclear TyrRS, mTOR inhibition can functionally replace the nuclear TyrRS to suppress translation. We again observed no difference in mTOR activity between *Yars*^+/+^ cells and *Yars*^ΔNLS/ΔNLS^ cells with or without the Torin treatment (Figure S8B). Therefore, although there may be a functional overlap between mTOR inhibition and nuclear TyrRS in controlling translation during chronic oxidative stress, nuclear TyrRS and mTOR act independently.

### Possible mechanism by which nuclear TyrRS regulates translation in HEK293 cells

**Figure 3.**
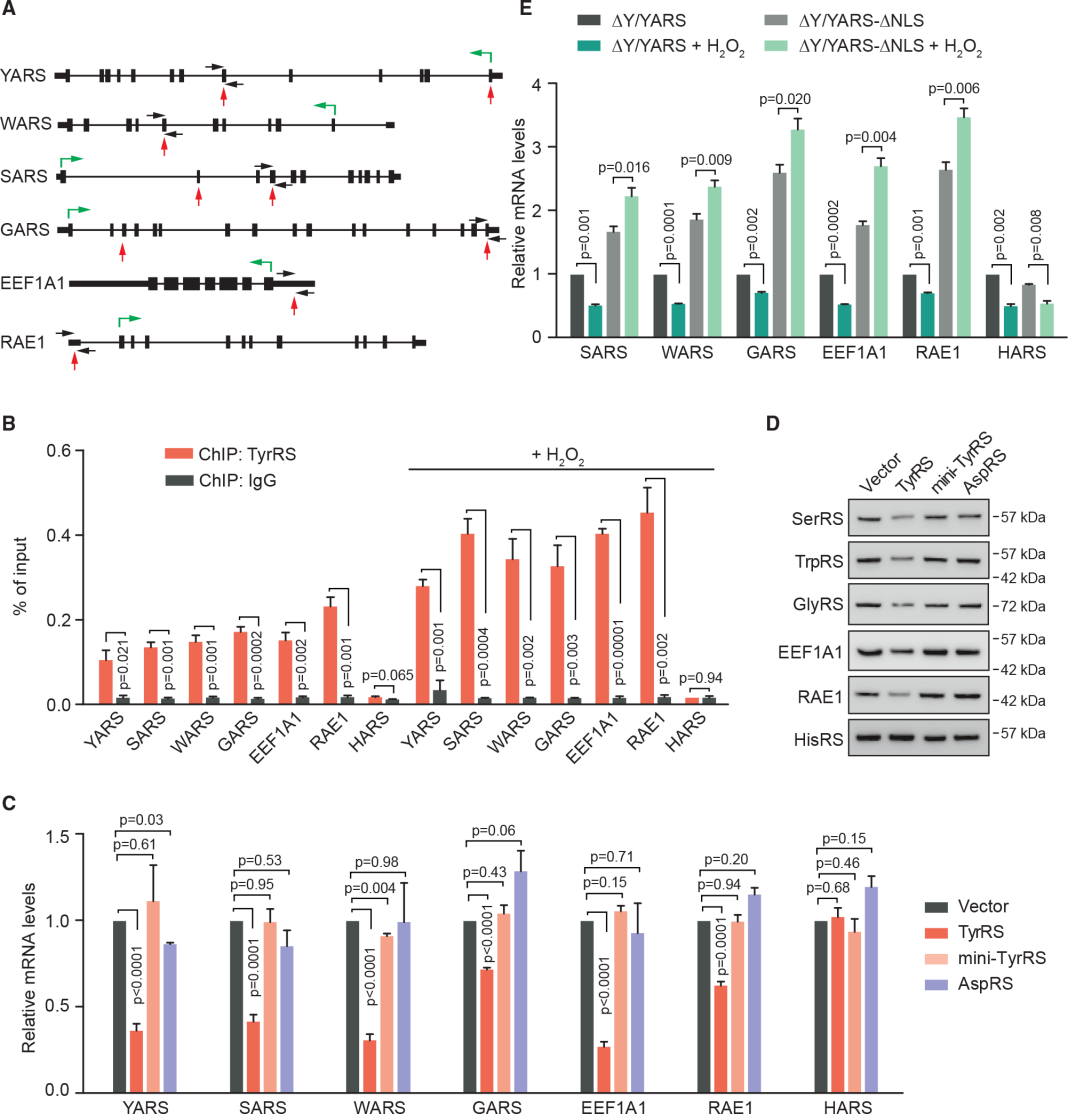
Nuclear TyrRS binds to DNA elements of translation-related genes and represses their transcription in HEK293 cells (A) Illustration of TyrRS-binding sites on its translation-related target genes as identified by ChIP-seq. Black boxes, exons. Black thick lines, 5′ or 3′ UTR. Black thin lines, introns. Red arrows, TyrRS-binding sites. Black arrows, primers for ChIP-qPCR. Green arrows, directions and starting sites of translation. (B) ChIP-qPCR confirms the DNA-binding ability of endogenous TyrRS on translation-related genes, which is enhanced with 12 h H_2_O_2_ treatment. n = 3, biological replicates, Student’s t test. (C) Overexpression of TyrRS (24 h) represses transcription of translation-related genes shown by RT-PCR. n = 3, biological replicates, Student’s t test. (D) Overexpression of TyrRS (48 h) downregulates target genes’ protein level shown by western blot. (E) Restricting TyrRS nuclear localization promotes transcription of translation-related genes especially after 12 h H_2_O_2_ treatment compared with transcriptional repression in control cells. n = 3, biological replicates, Student’s t test.

Using HEK293 cells stably expressing V5-tagged-TyrRS and chromatin immunoprecipitation (ChIP), we found a total of 20 DNA sites bound by TyrRS (Table S1). Many of these binding sites (16/20) fall within the coding region of these genes. Interestingly, six TyrRS-bound genes encode translation-related proteins, including TyrRS itself (YARS1) and three other tRNA synthetases (SARS1, WARS1, and GARS1), eukaryotic elongation factor (EEF1A1), and mRNA nuclear export gene (RAE1) (Figure 3A). Using ChIP-PCR analysis, we confirmed the binding of endogenous TyrRS in wild-type HEK293 cells to these six genes but not to a control tRNA synthetase gene, HARS1 (Figure 3B), and the binding was enhanced with H_2_O_2_ treatment as expected (Figure 3B). Expression of TyrRS inhibits the transcription of TyrRS target genes but not of the control gene HARS1, which was not bound by TyrRS (Figure 3C). In contrast, expression of human AspRS (a tRNA synthetase that we use as a control) or mini-TyrRS did not significantly affect the transcription of TyrRS target genes (Figures 3C and S2), consistent with their lack of impact on global translation (Figure 1C). The changes at the transcript level of these genes were consistent with the changes at the protein level (Figure 3D).

To confirm that the effect of TyrRS was not due to overexpression, we tested the transcription level of TyrRS target genes in ΔY/YARS and ΔY/YARS-ΔNLS HEK293 cells. Excluding TyrRS from the nucleus increased the transcription of SARS1, WARS1, GARS1, EEF1A1, and RAE1, but not HARS1 (Figure 3E), supporting the hypothesis that nuclear TyrRS at endogenous expression levels is able to repress the transcription of its target genes. Interestingly, in “normal” (ΔY/YARS) cells, 12 h H_2_O_2_ treatment significantly decreased the transcript level of TyrRS target genes. In contrast, the transcript levels of TyrRS target genes were significantly increased, rather than decreased, in nuclear TyrRS-deficient (ΔY/YARS-ΔNLS) cells under H_2_O_2_ treatment (Figure 3E). HARS1 (non-target gene) transcription was decreased in both “normal” and nuclear TyrRS-deficient cells after a 12 h H_2_O_2_ treatment (Figure 3E), suggesting that HARS transcription was also suppressed under oxidative stress but through a TyrRS-independent mechanism. These results correlate with the observation that nuclear TyrRS-deficient cells aberrantly activate translation (Figure 1A) and support the possibility that nuclear TyrRS inhibits global translation through transcriptionally repressing certain translation-related genes.

We were able to extract a consensus sequence (5′-CTCCTC-3′) from DNA-binding sites of nuclear TyrRS (Figure S9A). Purified recombinant human TyrRS could bind to a 28 bp DNA containing the consensus sequence *in vitro*, while mutating the consensus sequence from 5′-CTCCTC-3′ to 5′-CTTTTC-3′ abolished the interaction (Figure S9B). The DNA binding and the specificity of TyrRS were confirmed using HEK293 cells expressing TyrRS-V5. As shown in Figure S9C, the cell nuclear extract could cause a mobility shift of the 28 bp wild-type DNA oligonucleotide but not of the mutant DNA. Pre-incubating the nuclear extract with α-V5 antibodies, but not with an immunoglobulin G (IgG) control, caused a “super shift,” confirming that it was indeed TyrRS-V5 that bound to DNA (Figure S9C). Neither mini-TyrRS nor the evolutionarily new CTD alone could bind to the DNA, suggesting that both regions are required for DNA binding (Figures S9C and S9D).

Active genomic regulatory elements are often flanked by histones with H3 acetylation modification at lysine 27 residue (H3K27Ac).^39^ Overexpressing TyrRS in HEK293 cells reduced the H3K27Ac levels on TyrRS target genes (Figure S10A). Several histone deacetylases, including HDAC1, HDAC2, and HDAC3, were identified to interact with TyrRS in HeLa cells.^19^ The interaction between TyrRS and HDAC1 is not direct but is mediated by transcription co-repressor TRIM28.^19^ To confirm these interactions and to identify other potential interaction partners of TyrRS in HEK293 cells, we performed a stringent tandem affinity purification and mass spectrometry (TAP-MS) analysis with TyrRS fused with two high-affinity tags, a streptavidin-binding peptide (SBP), and a calmodulin-binding peptide (CBP) (Figure S11). The result confirmed HDAC2 and TRIM28 as TyrRS interactors in HEK293 cells and identified two other histone modification-related proteins, CHD4 and MBD3, as candidate interaction partners of TyrRS (Table S2). Intriguingly, CHD4, MBD3, HDAC2, and HDAC1 are all components of the nucleosome remodeling deacetylase (NuRD) complex, a major chromatin remodeling complex, which also contains other components, including RbAp46, RbAp48, CHD4, MBD3, MTA1, p66α, and LSD1. Using co-immunoprecipitation (coIP), we confirmed that TyrRS can interact with HDAC1, HDAC2, CHD4, MBD3, and two other factors of the NuRD complex, MTA1 and p66α, in HEK293 cells (Figure S10B). These results suggest that in the nucleus, TyrRS can recruit different co-repressors (TRIM28/HDAC1 or NuRD complex) to epigenetically regulate target genes transcription. Indeed, overexpression of TyrRS significantly increased the binding of HDAC1, the shared co-factor between TRIM28 and the NuRD complex, to all the translation-related TyrRS target genes (Figure S10C). In contrast, TRIM28 was recruited by TyrRS to three of its target genes (*YARS1, WARS1*, and *GARS1*) (Figure S10D), while CHD4 was recruited to a different gene set (*YARS1*, *SARS1*, *EEF1A1*, and *RAE1*) (Figure S10E). Interestingly, all translation-related TyrRS target genes were bound by either the NuRD complex (as represented by CHD4) or TRIM28, or both, while *YARS1* was the only gene bound by both. The importance of these repressor complexes was further strengthened by the effect of Trichostatin A (TSA), a selective inhibitor of the class I and II HDACs (including HDAC1),^40,41^ in blocking the activity of TyrRS in regulating the expression of target genes (Figure S12). Taken together, nuclear TyrRS may regulate protein synthesis by binding to certain translation genes and by recruiting TRIM28 and/or the NuRD complex to transcriptionally repress their expression.

## DISCUSSION

Through their evolutionarily conserved enzymatic function in charging tRNA with their cognate amino acid, aaRSs are essential for protein synthesis in all domains of life. In complex multicellular organisms, the functional landscape of aaRSs is expanded with broad regulatory functions.^42–44^ However, the connections between their classical enzymatic role and their non-enzymatic functions remain to be understood.^45^ TyrRS was the first aaRS shown to have regulatory roles with several of these roles occurring in the nucleus.^19,24,31^ Due to the location of its NLS, nuclear import of TyrRS can be regulated by the cognate tRNA.^21^ This regulation and the increasing evidence for the involvement of tRNA in translational control during stress^46,47^ suggested a role of TyrRS nuclear translocation in stress response. Indeed, we found that the nuclear import of TyrRS is stimulated under stress and that nucleus-localized TyrRS functions through the transcriptional machinery to promote the expression of DNA damage response genes for cell protection.^19^ Here, we reveal that nuclear TyrRS also inhibits global translation, possibly by binding to translation-related genes and recruiting TRIM28/HDAC1 or the NuRD complex to repress their transcription.

**Figure 4.**
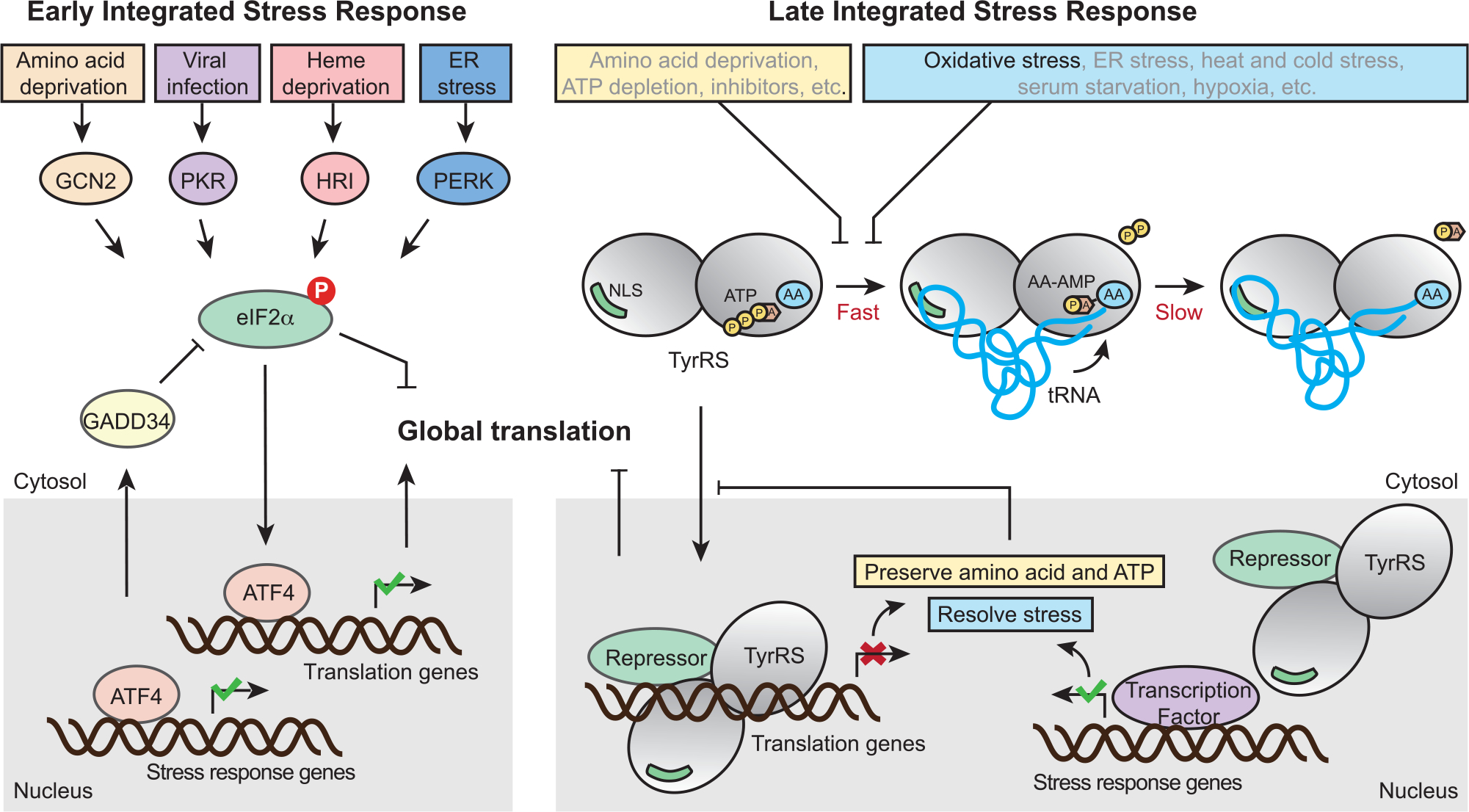
Schematic illustration of the classical ISR and the proposed TyrRS-mediated chronic “ISR” pathways

It has been demonstrated that a variety of stressors including UV exposure, heat stress, serum starvation, H_2_O_2_ (oxidative stress), sodium arsenite (oxidative stress), and tunicamycin (ER stress) treatment can trigger TyrRS nucleus translocation.^19,24^ Consistently, in addition to oxidative stress, we were able to confirm that ER stress also stimulates TyrRS nucleus translocation in HEK293T cells (Figure S13). We propose that these stress signals are transmitted through intrinsic properties of the tRNA synthetase enzyme. The aminoacylation reaction happens in two steps: first, TyrRS binds to tyrosine and ATP to catalyze the formation of an enzyme-bound tyrosyl-adenylate, which triggers tRNA binding for the second step: the activated amino acid subsequently reacts with tRNA to yield aminoacyl-tRNA (Figure 4). For most aaRSs, including TyrRS, the second step or the release of the aminoacyl-tRNA product is rate limiting.^48^ Therefore, in unstressed cells, most TyrRS in the cytosol exists in a tRNA-bound state in which the NLS is blocked (Figure 4). Depletion of tRNA leads to TyrRS NLS exposure and nuclear translocation.^21^ Under a variety of stress conditions, including oxidative stress, heat stress, UV exposure, serum starvation, cold stress, and hypoxia, tRNAs are cleaved by angiogenin.^23,49^ Depletion of tRNA can also result from retrograde nuclear tRNA import, which is also increased upon oxidative stress.^50^ Specifically, tRNA^Tyr^ is fragmented in response to oxidative stress, resulting in lower levels of mature tRNA.^51^ NLS exposure can also be achieved by modification of the synthetase, independent of the status of tRNA. A recent report showed that enhanced TyrRS nuclear localization during oxidative stress is mediated through acetylation at K244 within its NLS.^29^ Compared with the WT protein, we found that the acetylation mimicry mutant TyrRS^K244Q^ has a decreased binding affinity toward the cognate tRNA (Figure S14), which would increase the exposure of the NLS and lead to enhanced nuclear localization of TyrRS, as confirmed (Figure S15). Another mechanism to promote TyrRS nuclear translocation may be through depleting the substrates (i.e., ATP and amino acid), which would slow down the first step of the aminoacylation reaction, when the synthetase is free from tRNA binding (Figure 4). Indeed, inhibiting the enzymatic activity of TyrRS through resveratrol, a tyrosine-like molecule, was shown to stimulate the nuclear translocation of the synthetase.^24^ Therefore, through its active site, TyrRS has the intrinsic capability to sense amino acid and energy deficiency within the cell. Importantly, this type of metabolic stress can be integrated with tRNA-mediated stress stimuli through the nuclear translocation of TyrRS, subsequently leading to global translation inhibition and stress-response genes activation.

Similar to the negative feedback regulation instigated in ISR to prevent over-suppression of translation and to restore homeostasis, the tRNA synthetase-mediated response also provides negative feedback mechanisms: the stress response and translation inhibition affected by nuclear TyrRS would preserve ATP and amino acid and help alleviate the stressors that stimulate TyrRS nuclear translocation in the first place (Figure 4). In addition, TyrRS represses its own gene expression to ensure the repression is tapering off and not overdone. Interestingly, three other aaRS genes (i.e., WARS1, SARS1, and GARS1) were also targeted by nuclear TyrRS (Figure 3A), raising the possibility that TyrRS is not the only aaRS mediating this type of stress response. Presumably, having multiple aaRSs involved in the regulation would increase the breadth (e.g., detecting the status of additional amino acids and tRNA species) and the capacity for the stress response. Interestingly, all four aaRSs targeted by TyrRS are standalone and not associated with the multisynthetase complex,^52,53^ suggesting that mobility could be a required property for a synthetase to be involved in this stress response network. Future studies should investigate nuclear functions of these and other aaRSs in stress response and whether the tRNA-controlled nuclear translocation is a common scheme.

Nuclear TyrRS-mediated translational suppression occurred later than eIF2α phosphorylation (Figure 1A). The effect of H_2_O_2_ treatment on eIF2α phosphorylation was acute due to a negative feedback regulation through ATF4 and its target gene GADD34, which dephosphorylates eIF2α to reinstate protein synthesis (Figure 4). Indeed, we observed ATF4 activation in both normal and nuclear TyrRS-deficient cells under oxidative stress (Figure 1A). Interestingly, many translation genes suppressed by nuclear TyrRS, including *YARS1*, *WARS1*, *SARS1*, and *GARS1,* are also target genes of ATF4 with an opposite effect (activation) on their expression,^12,26^ suggesting that nuclear TyrRS counteracts ATF4. Indeed, excluding TyrRS from the nucleus not only abolished the oxidative stress-induced repression of its target genes but also led to an over-stimulation of these genes (Figure 3E), possibly mediated by the activated ATF4, no longer kept in check by nuclear TyrRS. Therefore, we suggest that the cellular ISR may constitute at least two stages, both aiming to adapt and then to restore. The classical eIF2α/ATF4-based ISR provides the acute response; if the stress is not yet resolved, the aaRS-based responses provide the cells with a second chance to adapt and survive through the stress condition. The ATF4-mediated activation of aaRSs may provide an internal connection and regulation between the two stages. However, whether regulating the 6 translation-related genes we identified is sufficient to account for the translation inhibition effect of nuclear TyrRS in HEK293 cells and whether there are cell-type-specific mechanisms by which nuclear TyrRS regulates translation remain important open questions. Nevertheless, based on our observations that excluding TyrRS from the nucleus aberrantly activated translation in both human HEK293 cells and mouse fibroblasts, the role of nuclear TyrRS in translation regulation is likely to be a conserved function in mammalian cells.

### Limitations of the study

Most of our mechanistic studies are based on the use of engineered HEK293 cells. Although the nuclear TyrRS-mediated stress response and the translational control is likely conserved in mammalian cells, it is unclear whether the specific mechanism of regulation is conserved. There is also a lack of evidence at the protein level to support the mechanism of regulation. Given the selective regulation of nuclear TyrRS on certain aaRS genes, it is interesting to ask whether, in addition to inhibiting global translation, the nuclear translocation of TyrRS would also alter the translation of a specific subset of transcripts, leading to a remodeled proteome. A combination of RNA sequencing (RNA-seq), ribosome profiling, and proteomic analyses would help to address these questions in the future.

## STAR★METHODS

### RESOURCE AVAILABILITY

#### Lead contact

Further information and requests for resources and reagents should be directed to and will be fulfilled by the lead contact, Xiang-Lei Yang (xlyang@scripps.edu)

#### Materials availability

All reagents generated in this study are available upon request from the lead contact.

#### Data and code availability

- CHIP-Seq data have been deposited at GEO and are publicly available as of the date of publication. Accession number is listed in the key resources table.
- This paper does not report original code.
- Any additional information required to reanalyze the data reported in this paper is available from the lead contact upon request.

### EXPERIMENTAL MODEL AND SUBJECT DETAILS

#### Animal models

The ΔNLS mice were generated by altering exon 7 of the Yars1 gene (that contains the NLS sequence) from ^242^KKKLKK^247^ to ^242^NNKLNK^247^. An FRT-flanked Neomycin cassette was introduced in the adjacent intron that was later bred out to create the ΔNLS-Neo-Out strain. mRNA expression of the mutant allele was confirmed through RT-PCR, and functional disruption of nuclear import was confirmed by Western blot. These F0 mice were crossed with C57BL/6J mice to obtain *Yars*^+/ΔNLS^ mice that were used for breeding thereafter. Female and male mice were used for breeding from ages 4–8 months of age for MEF generation. Mouse experiments were approved by and conducted in accordance with the guidelines of The Scripps Research Institute IACUC.

#### Cell lines and primary cultures

HEK-293 (human embryonic kidney) and HEK-293T cells were purchased from ATCC. HEK-293 and HEK-293T were grown in DMEM (Gibco) supplemented with 10% heat-inactivated fetal bovine serum (FBS, Gibco) and penicillin/streptomycin (Gibco). Cultures were maintained at 37C in a humidified atmosphere under 5% CO_2_.

HEK293 cells were transfected with constructs pEco-Lenti-H1-shYARS-YARS or pEco-Lenti-H1-shYARS-YARS-ΔNLS^19^ which contain shRNA targeting 3′UTR of human YARS mRNA and coding sequence of human WT TyrRS or NLS-mutated (^242^NNKLNK^247^) TyrRS. About 48 h after transfection, cells were selected with 1 μg/ml puromycin for 10 days to generate ΔY/YARS and DY/ YARS-ΔNLS cells.

Primary MEFs were isolated from heterozygous, *Yars*^+/ΔNLS^, mice as previously described.^57^ Briefly, embryos were collected between E12.5 – E14.5 from pregnant (*Yars*^+/ΔNLS^ *x Yars*^+/ΔNLS^) females that were dissected out of uterine horns into PBS with 1% penicillin/streptomycin. The embryos were separated from the placenta, and the head and organs were removed, then the remaining blood was rinsed off. The cells were isolated from trypsinized embryonic tissue. Cells from each embryo were plated in a 10 cm^2^ dish with media containing DMEM with Glutamax (Gibco), 10% heat-inactivated embryonic stem cell fetal bovine serum (ThermoFisher, 16141079), and 1% penicillin/streptomycin (Gibco). Primary MEFs were maintained in a 37°C incubator at 5% CO_2_, and all experiments were conducted at passages 1–3. Tissue from embryos were saved for genotyping. DNA was extracted using Lucigen QuickExtract (VWR, 76081–766), and the knock-in alleles were detected by PCR amplification with the primers below:

Forward: ATAGGTTCTTCCTGCAGGGAC.

Reverse: TCTGCCTCTAGTCTCATTTTGGATTC.

TyrRS mutant B16-F10 cell lines were created by the combination of Tet-on inducible system and the CRISPR-Cas9 technology. Mouse Yars cDNA was cloned into pRetroX-TetOne-Puro vector (Clonetech). Mouse B16-F10 melanoma cells were transfected with 5 mg of pRetroX-TetOne-Puro containing the mouse Yars sequence (fused to a C-terminal His_6_ tag to be differentiated from the endogenous TyrRS) with the silent PAM mutation (AGG→AAG) using xFect (Takara Bio) according to the manufacturer’s instruction. After 24 h, transfected cells were selected by adding 2 μg/mL of puromycin (Sigma Aldrich). The B16-F10 cells were further transfected with pCRISPR-CG01 (GeneCopoeia) containing the sgRNA sequence which targeted exon 7 of the mouse Yars gene. The knockouts of the Yars gene were confirmed by DNA sequencing of PCR fragments spanning the target sites. The lack of the endogenous TyrRS protein expression was also verified by western blotting using an anti-TyrRS antibody (Santa Cruz Biotechnology). During the knockdown period, the cell survival was maintained by the expression of TyrRS from pRetroX-TetOne-Puro vector induced by 100 ng/mL of doxycycline. This cell line was further transfected with modified pCDNA3.1 vector encoding WT, K244Q, or K244R Yars cDNA fused to GFP. Cells expressing TyrRS-GFP of WT, K244Q, or K244R could be selected by removal of doxycycline and the expression of TyrRS-GFP proteins was confirmed by flow cytometry and western blotting.

### METHOD DETAILS

#### Cell treatment

All treatments were conducted at 70–80% confluence in HEK293 cells. Transfections were performed using LipofectAMINE 2000 (Life Technology) according to the manufacturer’s instructions. For H_2_O_2_ (Millipore Sigma, H1009) treatment, H_2_O_2_ was diluted in PBS and added into the cell media to a final concentration of 200 μM for the indicated period of time. For homoharringtonine (HHT) treatment (Millipore Sigma), HHT was added into cell media to a final concentration of 50 mM. For Trichostatin A (TSA, Millipore Sigma) treatment, TSA was added into cell culture media to a final concentration of 0.3 μM for 24 h. For both HHT and TSA treatment, DMSO was used as the control. For the E2F1knock down experiment, cells were transfected with shControl (Santa Cruz Biotechnology) or shE2F1 (Santa Cruz Biotechnology) using LipofectAMINE 2000, 72 h prior to the experiments. Mouse embryonic fibroblasts were grown to 50–70% confluency before treatments. Homoharringtonine (Cayman Chemical), Cycloheximide (Fisher scientific), and Torin-1 (Fisher Scientific) were solubilized in DMSO and used at 50 nM, 50 ng/mL, or 31.25 nM, respectively. Hydrogen peroxide (Millipore Sigma) was applied at 200 μM, and tunicamycin (Cayman Chemical) was applied at 5 μg/mL. All chemicals used were listed in key resources table.

#### Surface sensing of translation (SUnSET)

Each sample was one well of a 6-well plate, estimated around 300,000 cells per sample. Cells with the indicated treatment were incubated with 10 mg μL^−1^ of puromycin (Sigma Aldrich) for 30 min. After treatment, cells were washed twice in PBS, suspended in lysis buffer (Cell Signaling), and analyzed by Western blot with an antibody against puromycin (1:5000). α-tubulin (1:3000) was used as a loading control. The amount of newly synthesized protein was quantified using the ProteinSimple Western Blot analysis software and normalized to α-tubulin signal.

#### Western blot assay

Each sample was one well of 6-wells plate (except Co-IP experiments), estimated around 300,000 cells per sample. Cells were washed twice in phosphate-buffered saline (PBS), collected, suspended in lysis buffer (Cell Signaling) for 30 min, and centrifuged for 7 min at 12,000 *g; The soluble lysates were then collected, fractionated by SDS-PAGE, and transferred to PVDF membranes using the iBlot Dry Blotting System (Life Technologies). Membranes were blocked for 1 h with Tris-Buffered Saline with Tween 20 (TBST) containing 5% nonfat dry milk. The following primary antibodies were diluted in 1% milk in TBST prior to usage at the indicated concentration: mouse monoclonal α-tubulin (1:3000), rabbit monoclonal p-p70 S6K (1:1000), rabbit monoclonal p-4E-BP1 (1:1000), rabbit monoclonal p70 S6K (1:1000), rabbit monoclonal 4E-BP1 (1:1000), rabbit polyclonal p-eIF2α (1:1000), rabbit polyclonal eIF2α (1:1000), rabbit monoclonal ATF-4 (1:1000), rabbit polyclonal SARS (1:1000, made in-house), rabbit polyclonal WARS1 (1:3000, made in-house), mouse monoclonal GARS (1:4000, made in-house), rabbit monoclonal EEF1A1 (1:1000), goat polyclonal RAE1 (1:1000), rabbit polyclonal HARS (1:1000), mouse monoclonal V5 (1:5000), mouse monoclonal HDAC1 (1:1000), mouse monoclonal HDAC2 (1:1000), mouse monoclonal HDAC3 (1:1000), mouse monoclonal RbAp46 (1:1000), mouse monoclonal RbAp48 (1:1000), mouse monoclonal CHD4 (1:1000), rabbit monoclonal MBD3 (1:1000), rabbit polyclonal MTA1 (1:1000), rabbit polyclonal p66a (1:1000), rabbit polyclonal LSD1 (1:1000), rabbit polyclonal TRIM28 (1:1000), rabbit polyclonal caspase 3 (1:1000), rabbit polyclonal cleaved caspase 3 (1:1000), rabbit monoclonal PARP (1:1000), rabbit polyclonal SOD1 (1:1000), rabbit polyclonal Catalase (1:1000), rabbit polyclonal Thioredoxin (1:1000). After incubation with primary antibodies, the membranes were washed and incubated with HRP-conjugated anti-mouse or anti-rabbit secondary antibodies (1:3000) and followed by detection with ECL Western blot substrate (Fisher Scientific). Primary and secondary antibodies used for western blotting are listed in the key resources table.

#### Chromatin immunoprecipitation (ChIP) assay

The ChIP-seq experiment was performed with an α-V5 antibody and a HEK293 cell line that stably expresses V5-tagged-TyrRS. A stable HEK293 cell line with only V5-tag expression was used as a control. Each sample was one 10 cm dish, estimated around 6,000,000 cells per sample. Cells were fixed with formaldehyde (1% final concentration) for 10 min at room temperature. The reaction was stopped by adding 125 mM glycine. ChIP assays were performed according to the protocol of ChIP-IT Express Enzymatic kit (Active Motif) using ChIP-grade antibody against V5 (Fisher Scientific), IgG (Cell signaling technology), H3K27Ac (Abcam), HDAC1 (Abcam), TRIM28 (Abcam), or CHD4 (Abcam). In-house made TyrRS antibody was used to pull down endogenous TyrRS. The eluted DNA fragments were subjected to library preparation and sequencing or analyzed *via* a StepOnePlus Real-time PCR system using SYBR Select Master Mix (Applied Biosystems). For ChIP-qPCR, the primer sets targeting different genes were as follows: YARS ChIP (forward): 5′-TGAATTACAGTCACTGTCAAAGCTACAT-3′; YARS ChIP (reverse): 5′-AATAGCCAAGTGCAGGGAGGTACTGAGA-3′; SARS ChIP (forward): 5′-GGCAACCGAATCATATTCCTGTG-3′; SARS ChIP (reverse): 5′-CTCAAACCGCTCTGCTTCCAACT-3′; WARS ChIP (forward): 5′-GAGGGAGTGAATTTCTTTGGTTGG-3′; WARS ChIP (reverse): 5′-GCAGCAGGGCTGGTTTAGGATAG-3′; GARS ChIP (forward): 5′-CTGGCATGACATTGTTTGGCTCTGTG-3′; GARS ChIP (reverse): 5′-CAGAGGATACCTGGCCTCCACAT CAG-3′; HARS ChIP (forward): 5′-ACATTACAGGCATGAGCCACC-3′; HARS ChIP (reverse): 5′-CTAGCTGATTGTGATAAGAGAGA GAACAGC-3′; EEF1A1 ChIP (forward): 5′-AGTTGCGTGAGCGGAAAGATG-3′; EEF1A1 ChIP (reverse): 5′-CTAATCGAGGTGCCTG GACGG-3′; RAE1 ChIP (forward): 5′-GGGGCGGTTCACGATGTT-3′; RAE1 ChIP (reverse): 5′-CAGCAGTTTGCGAATTGGTC-3′. The signal from the ChIP samples was quantified as percentage of input relative to the whole cell lysate.

#### TyrRS-chip-seq

Each sample size was five 10 cm dishes, estimated around 10,000,000 cells per sample. Libraries were prepared and sequenced by the Next Generation Sequencing Core at Scripps Research, La Jolla, California. Sequencing depth (reads): V5 repeat 1: 20,567,024 (73.39% overall alignment to hg19), V5 repeat 1 input: 22,802,577 (92.61% overall alignment), V5 repeat 2: 15,358,168 (80.43% overall alignment), V5 repeat 2 input: 23,228,662 (93.04% overall alignment), IgG control: 13,431,892 (78.67% overall alignment), IgG control input: 29,488,182 (89.09% overall alignment). Reads were quality filtered and adapter trimmed. In brief, the ENCODE 3 ChIP-Seq pipeline^58^ was followed for read mapping and peak calling: reads were aligned to hg19 with Bowtie2^55^ and duplicates were removed using picard tools and samtools.^56^ TyrRS binding sites in the genome in comparison to a control input were identified using MACS.^59^ The data is deposited at GEO and the accession number is listed in the key resources table.

#### Real-time PCR

Each sample was one well of 6-wells plate, estimated around 300,000 cells per sample. Total RNAs were extracted from pre-treated cells using Trizol (Life Technologies) according to the manufacturer’s instructions and then reverse-transcribed using the first strand cDNA synthesis kit (Fisher Scientific). The cDNA products were analyzed on a StepOnePlus Real-time PCR system using SYBR Select Master Mix (Applied Biosystems). The primers used for different target genes are: YARS RT (forward): 5′-CAACCTGGCTG GACTCGCGTGACAGTTC-3′; YARS RT (reverse): 5′-AGGTTCCGGGTGATAAGGTGCAGTTTCT-3′; SARS RT (forward): 5′-GGAACC TTCTGCACCCTTCTGTACCCAT-3′; SARS RT (reverse): 5′-TTTCGCCTTCAAAGCCATCTACCATCAC-3′; WARS RT (forward): 5′-CA AGGACATCATCGCCTGTGGCTTTGAC-3′; WARS RT (reverse): 5′-CTGTGGGAATGAGTTGCTGAAGGAGGGA-3′; GARS RT (forward): 5′-CTGTAGTTGCTCCATTCAAATGTTCCGTCCTC-3′; GARS RT (reverse): 5′-TCTCATCAGTCCTGGCATAGCGCCTTCC-3′; HARS RT (forward): 5′-GAAGAATGAGATGGTGGGAGAGA-3′; HARS RT (reverse): 5′-CAATGCCAAATAGGGTCAGGTA-3′; EEF1A1 RT (forward): 5′-ACACGTAGATTCGGGCAAGTCCACCACT-3′; EEF1A1 RT (reverse): 5′-TGATACCACGTTCACGCTCAGC TTTCAG-3′; RAE1 RT (forward): 5′-TGTGATGACTGGGAGCTGGGATAAGACT-3′; RAE1 RT (reverse): 5′-CACCGATGCTGATGTTTC AGTGGAGATT-3′.

#### Nuclear-cytoplasmic fractionation

Each sample was one 6 cm dish, estimated around 500,000 cells per sample. Nuclear-cytoplasmic fractionation was conducted using the NE-PER Nuclear and Cytoplasmic Extraction Reagents kit (Thermo Scientific) according to the manufacturer’s protocol.

#### TAP-mass spec

InterPlay Mammalian TAP System was purchased from Agilent. Human TyrRS (GenBank: NM_003680.3) CDS was inserted into the Protein Interactions Expression Vector provided by the system to generate constructs to express TyrRS with SBP and CBP tags. The construct was transfected into HEK293 cells for 48 h, cells were lysed and spun down, and whole cell lysate was added to the Streptavidin resin for SBP tag binding. The elution from the Streptavidin resin was next added to the Calmodulin resin for CBP tag binding. The product from the final elution and whole cell lysate were subjected to silver staining using Silver Stain Plus Kit (BIO-RAD) to check for pull down efficiency and purity. The eluted proteins were analyzed using the methods and protocols described in Pankow et al.^60^ and Pankow et al.^61^

#### Co-immunoprecipitation (Co-IP)

Each sample was one 10 cm dish, estimated around 2,000,000 cells per sample. HEK293 cell lysates were prepared as described above for Western blot analysis. Protein G beads (Life Technologies) were pre-incubated with anti-TyrRS antibody for 30 min and then mixed with cell lysates overnight. The beads were then washed 3 times with buffer (100 mM NaCl, 50 mM Tris, pH 7.5, 0.1% Triton X-100, 5% glycerol), and immunoprecipitated proteins were analyzed by SDS-PAGE and Western blot with the indicated antibodies.

#### Cell viability assay

Each sample was one well of 96-wells plate, estimated around 10,000 cells per sample. Relative cell viability was determined using a Cell counting kit-8 (Dojindo) and read on a microplate reader (Promega).

#### Cellular ROS analysis

Each sample was one well of a 96-well plate, estimated around 10,000 cells per sample. Cells were loaded with CM-H_2_DCFDA (Invitrogen) for 30 min according to the manufacture’s instruction. Signals were read on a microplate reader (Promega).

#### Quantitative image analysis of nuclear localization of TyrRS and mutants

The B16-F10 cells (WT, K244Q, K244R) were seeded on the sterilized cover slips coated with 25 μg/mL of Polyethylenimine (Polysciences). The cells were fixed with 2% paraformaldehyde for 30 min, and then the nuclei were stained with 20 μM of DRAQ5 (Fisher Scientific). The cover slips were mounted with Prolong Gold (Fisher Scientific). The images were acquired with a LSM880 laser scanning confocal microscope (Zeiss). All images were acquired using the same settings and conditions. The image analysis was performed with Fiji software (National Institute of Health).

#### Flow cytometry

B16-F10 cells (WT, K244Q, K244R) were detached using 0.25% trypsin-EDTA, and samples were analyzed using Novocyte flow cytometer (ACEA Biosciences). The data were analyzed using FlowJo software (FlowJo LCC).

#### Filter binding assay

Human tRNA^Tyr^ transcript (HGNC symbol: TRY-GTA2–1) was transcribed overnight at 37°C using the HiScribe T7 Quick High Yield RNA Synthesis Kit (New England Biolabs) according to the manufacturer’s protocol. The reaction was gel purified using a Novex TBE-Urea gel (Fisher Scientific) followed by ethanol precipitation. The purified transcript was labeled at the 5′-end with [γ−^32^P]ATP using the KinaseMax kit (Fisher Scientific) and incubated with an increasing concentration gradient of recombinant human TyrRS (WT and K244Q) from 125 nM to 8 μM in 10 mM HEPES, pH 7.5, 4 mM KCl, 0.02 mg/mL BSA, and 0.2 mM DTT for 30 min at RT. Each reaction was repeated in triplicate and samples were applied to nitrocellulose and nylon membranes using a Bio-Dot microfiltration apparatus (Bio-Rad) according to methods described in Rio.^62^ Membranes were washed two times with 60 mM HEPES, pH 7.5, and 10 mM MgCl_2_ to remove the unbound tRNA. Membranes were then exposed to a phosphorscreen overnight which was then scanned with a Typhoon imager (Cytiva). The fraction of TyrRS bound tRNA was quantified from the scan with ImageJ^54^ and plotted using GraphPad Prism (GraphPad Software).

### QUANTIFICATION AND STATISTICAL ANAYLSIS

GraphPad Prism 9 was used for data analysis and statistical significance was calculated using unpaired Student’s t-test or one-way ANOVA. Statistically significant differences are indicated in figures legends with the accompanying p values. Error bars in figures indicate the standard error of the mean (SEM) for the number of replicates (n), as indicated in the figure legend. p < 0.05(*), p < 0.01(**), p < 0.001(***), and p < 0.0001(****) were considered significant.

## Supplementary Material

1

2

3

## Figures and Tables

**KEY RESOURCES TABLE:** 

REAGENT or RESOURCE	SOURCE	IDENTIFIER

Antibodies		

Puromycin	Millipore Sigma	Catalog #: MABE343; RRID:AB_2566826
α-Tubulin	Cell Signaling Technology	Catalog #: 3873; RRID:AB_1904178
p-p70 S6K	Cell Signaling Technology	Catalog #: 9234; RRID:AB_2269803
p-4E-BP1	Cell Signaling Technology	Catalog #: 2855; RRID:AB_560835
p70 S6K	Cell Signaling Technology	Catalog #: 2708; RRID:AB_390722
4E-BP1	Cell Signaling Technology	Catalog #: 9644; RRID:AB_2097841
p-eIF2α	Cell Signaling Technology	Catalog #: 9721; RRID:AB_330951
eIF2α	Cell Signaling Technology	Catalog #: 9722; RRID:AB_2230924
ATF-4	Cell Signaling Technology	Catalog #: 11815; RRID:AB_2616025
EEF1A1	Cell Signaling Technology	Catalog #: 3586; RRID:AB_2096963
RAE1	Abcam	Catalog #: ab36139; RRID:AB_2175345
HARS	Abcam	Catalog #: ab137591
V5	Fisher Scientific	Catalog #: R960CUS; RRID:AB_2792973
HDAC1	Cell Signaling Technology	Catalog #: 5356; RRID:AB_10612242
HDAC2	Cell Signaling Technology	Catalog #: 5113; RRID:AB_10624871
HDAC3	Cell Signaling Technology	Catalog #: 3949; RRID:AB_2118371
RbAp46 (OTI5A4)	Novus Biologicals	Catalog #: NBP2–01308; RRID:AB_2725199
RbAp48 (13D10)	Novus Biologicals	Catalog #: NBP1–41201; RRID:AB_2238239
CHD4	Abcam	Catalog #: ab70469; RRID:AB_2229454
MBD3	Abcam	Catalog #: ab157464
MTA1	Abcam	Catalog #: ab71153; RRID:AB_1269482
P66a	Novus Biologicals	Catalog #: NB100-56643; RRID:AB_2294492
LSD1	Abcam	Catalog #: ab17721; RRID:AB_443964
TRIM28	Abcam	Catalog #: ab10483; RRID:AB_297222
Caspase 3	Cell Signaling Technology	Catalog #: 9662; RRID:AB_331439
Cleaved caspase 3	Cell Signaling Technology	Catalog #: 9661; RRID:AB_2341188
PARP	Cell Signaling Technology	Catalog #: 9532; RRID:AB_659884
SOD1	Abcam	Catalog #: ab13498; RRID:AB_300402
Catalase	Abcam	Catalog #: ab16731; RRID:AB_302482
Thioredoxin	Abcam	Catalog #: ab26320; RRID:AB_778412
anti-mouse HRP-conjugated	Cell Signaling Technology	Catalog #: 7076; RRID:AB_330924
anti-rabbit HRP-conjugated	Cell Signaling Technology	Catalog #: 7074; RRID:AB_2099233
IgG	Cell Signaling Technology	Catalog #: 5415; RRID:AB_10829607
H3K27Ac	Abcam	Catalog #: ab4729; RRID:AB_2118291
HDAC1	Abcam	Catalog #: ab7028; RRID:AB_305705
TyrRS Antibody (H-11)	Santa Cruz Biotechnology	Catalog #: sc-166741; RRID:AB_2219253
Beta Actin	Cell Signaling Technology	Catalog #: 4967; RRID:AB_330288

Chemicals, peptides, and recombinant proteins		

Homoharringtonine	Millipore Sigma	Catalog #: SML1091
Homoharringtonine	Cayman Chemical	Catalog #: 14631
Trichostatin A	Millipore Sigma	Catalog #: T1952
Cycloheximide	Fisher Scientific	Catalog #: AAJ6690103
Torin-1	Fisher Scientific	Catalog #: NC0418592
Hydrogen peroxide	Millipore Sigma	Catalog#: H1009
Tunicamycin	Cayman Chemical	Catalog#: 11445
Trizol	Life Technologies	Catalog #: 15596026
SYBR Select Master Mix	Applied Biosystems	Catalog #: 4472908
Puromycin	Sigma Aldrich	Catalog #: P9620

Critical commercial assays		

ChIP-IT Express Enzymatic kit	Active Motif	Catalog #: 53009
NE-PER Nuclear and Cytoplasmic Extraction Reagents	Thermo Scientific	Catalog #: 78833
Silver Stain Plus Kit	BIO-RAD	Catalog#: 1610449
Cell Counting Kit-8	Dojindo	Catalog #: CK04–01
CM-H2DCFDA	Invitrogen	Catalog #: C6827

Deposited data		

CHIP-Seq data	This paper	GEO: GSE155309

Experimental models: Cell lines		

HEK-293	ATCC	CRL-1573
HEK-293T	ATCC	CRL-3216
Primary Mouse embryonic fibroblasts	This paper	N/A
B16-F10	ATCC	CRL-6475

Experimental models: Organisms/strains		

Mouse: Yars^+/ΔNLS^ C57BL/6J	This paper	N/A

Recombinant DNA		

shControl	Santa Cruz Biotechnology	Catalog #: sc-108060
shE2F1	Santa Cruz Biotechnology	Catalog #: sc-29297-SH
InterPlay Mammalian TAP System	Agilent	Catalog#: 240101

Software and algorithms		

Graphpad Prism v9	GraphPad Software, LLC	https://www.graphpad.com/scientific-software/prism/
ImageJ	Schneider et al.^54^	https://imagej.nih.gov/ij/
Bowtie2	Langmead and Salzberg^55^	http://bowtie-bio.sourceforge.net/bowtie2/index.shtml
Samtools	Li et al.^56^	http://bowtie-bio.sourceforge.net/bowtie2/index.shtml
FlowJo	Becton, Dickinson and Company	https://www.flowjo.com/solutions/flowjo

## Highlights

- Nuclear TyrRS inhibits global translation during late-stage oxidative stress
- The effect of nuclear TyrRS is independent from the mTOR and ISR pathways
- Nuclear TyrRS is protective against cell death and ROS under oxidative stress
- Nuclear TyrRS acts by transcriptionally repressing translation-related genes
