# Supplementary material for: Nuclear translocation of an aminoacyl-tRNA synthetase may mediate a chronic “integrated stress response”
Source: Cell Rep. Author manuscript; Available in PMC 2023 Oct 23. (PMC10592355; doi:10.1016/j.celrep.2023.112632)
Supplement: 1 [file NIHMS1916159-supplement-1.pdf]

Cell Reports, Volume *42*

Supplemental information

Nuclear translocation of an aminoacyl-tRNA synthetase may mediate a chronic "integrated stress response"

Julia A. Jones, Na Wei, Haissi Cui, Yi Shi, Guangsen Fu, Navin Rauniyar, Ryan Shapiro, Yosuke Morodomi, Nadine Berenst, Calin Dan Dumitru, Sachiko Kanaji, John R. Yates III, Taisuke Kanaji, and Xiang-Lei Yang

Cell Reports, Volume ■■

Supplemental information

Nuclear translocation of an aminoacyl-tRNA synthetase may mediate a chronic "integrated stress response"

Julia A. Jones, Na Wei, Haissi Cui, Yi Shi, Guangsen Fu, Navin Rauniyar, Ryan Shapiro, Yosuke Morodomi, Nadine Berenst, Calin Dan Dumitru, Sachiko Kanaji, John R. Yates III, Taisuke Kanaji, and Xiang-Lei Yang

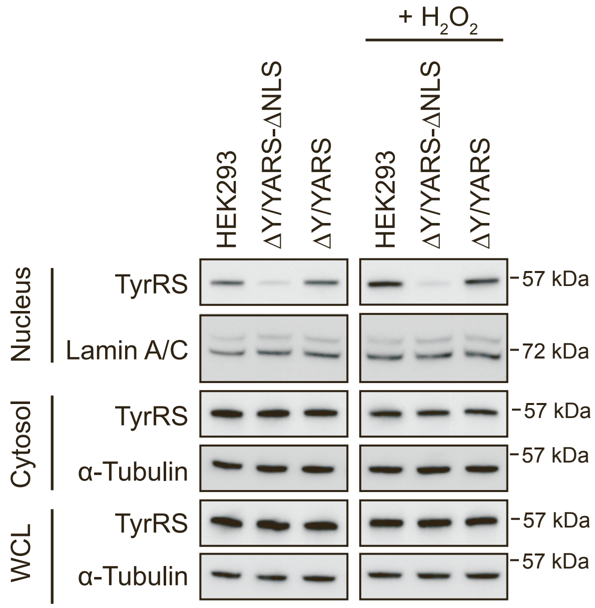

Figure S1: Cell fractionation and Western blot analysis to show that the expression level of TyrRS in ΔY/YARS and ΔY/YARS-ΔNLS cells are like that of the endogenous TyrRS in the original, unmodified HEK293 cells and that ΔY/YARS-ΔNLS cells are deficient in nuclear TyrRS with or without H_2O_2 treatment (12 hours). ΔY/YARS-ΔNLS: HEK293 cells with a knock down of endogenous TyrRS and expression of TyrRS with a mutated NLS (^{242}KKKLKK^{247} to ^{242}NNKLNK^{247}). ΔY/YARS: HEK293 cells with a knock down of endogenous TyrRS and ectopic expression of wild-type TyrRS. Lamin A/C: nuclear marker, α-Tubulin: cytoplasmic marker. WCL: whole cell lysate.

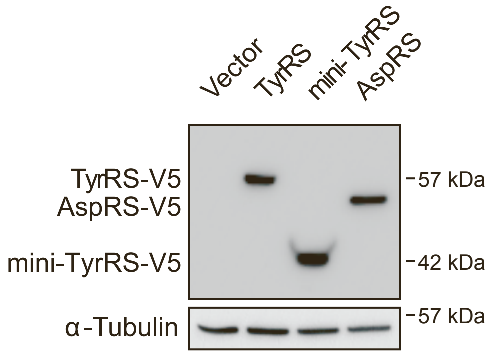

Figure S2: Western blot analysis to show the expression level of the V5-tagged TyrRS, mini-TyrRS and AspRS in HEK293 cells overexpressing each construct. Mini-TyrRS: Catalytic and tRNA binding domain of TyrRS. α-Tubulin: loading control.

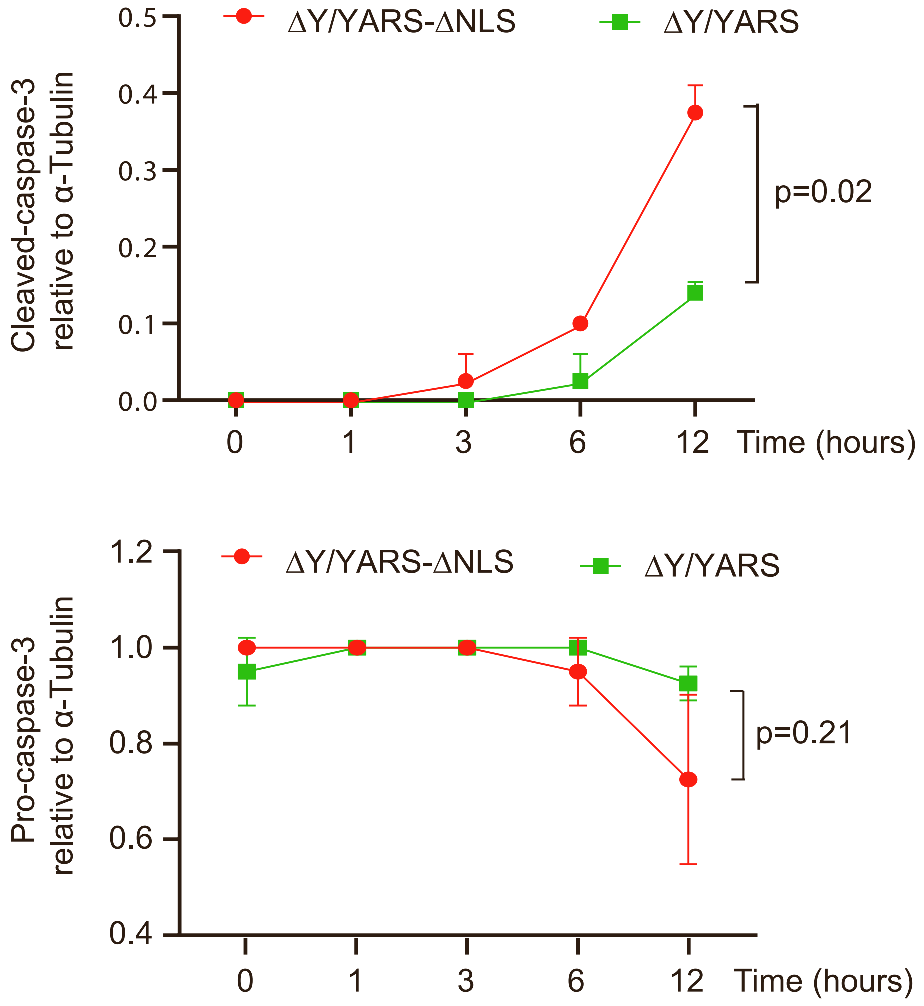

Figure S3: Quantification of caspase-3 cleavage as detected by western blot analysis shown in Figure 2B. n=2, biological replicates, Student's t-test.

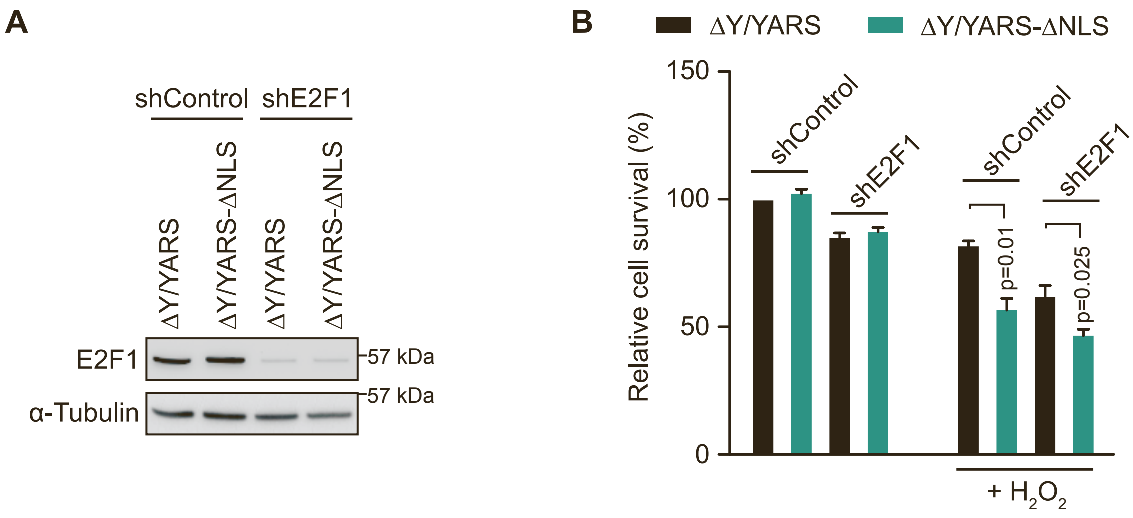

Figure S4: Nuclear TyrRS protects against cell death independent from its DNA damage protection effect, which is mediated by the transcription factor E2F1. ΔY/YARS-ΔNLS: HEK293 cells with a knock down of endogenous TyrRS and expression of TyrRS with a mutated NLS (^{242}KKKLKK^{247} to ^{242}NNKLNK^{247}). ΔY/YARS: HEK293 cells with a knock down of endogenous TyrRS and ectopic expression of wild-type TyrRS.

A) Western blot analysis confirms the knock down of shE2F1 in ΔY/YARS and ΔY/YARS-ΔNLS cells.

B) Stronger resistance to oxidative stress (H_2O_2 treatment for 36 hours) in cells with nuclear TyrRS (ΔY/YARS) compared to nuclear TyrRS-deficient cells (ΔY/YARS-ΔNLS) with or without E2F1 knockdown, indicating the protective effect of nuclear TyrRS against cell death is independent of E2F1. Relative cell viabilities were measured with a cell counting kit and viability of ΔY/YARS seeded at the same time but without H_2O_2 treatment was set at 100%. n=3, biological replicates, Student's t-test.

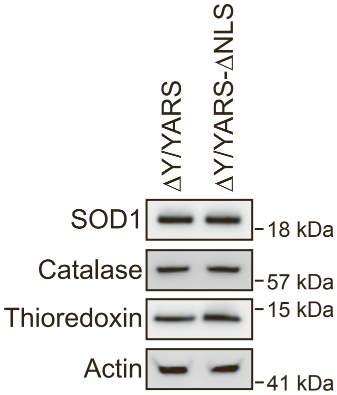

Figure S5: Nuclear TyrRS prevents cellular ROS over-accumulation independent of altering levels of common antioxidative stress response proteins SOD1, Catalase, and Thioredoxin. Cells were treated with H_2O_2 for 36 hours followed by western blot analysis. Actin: loading control.

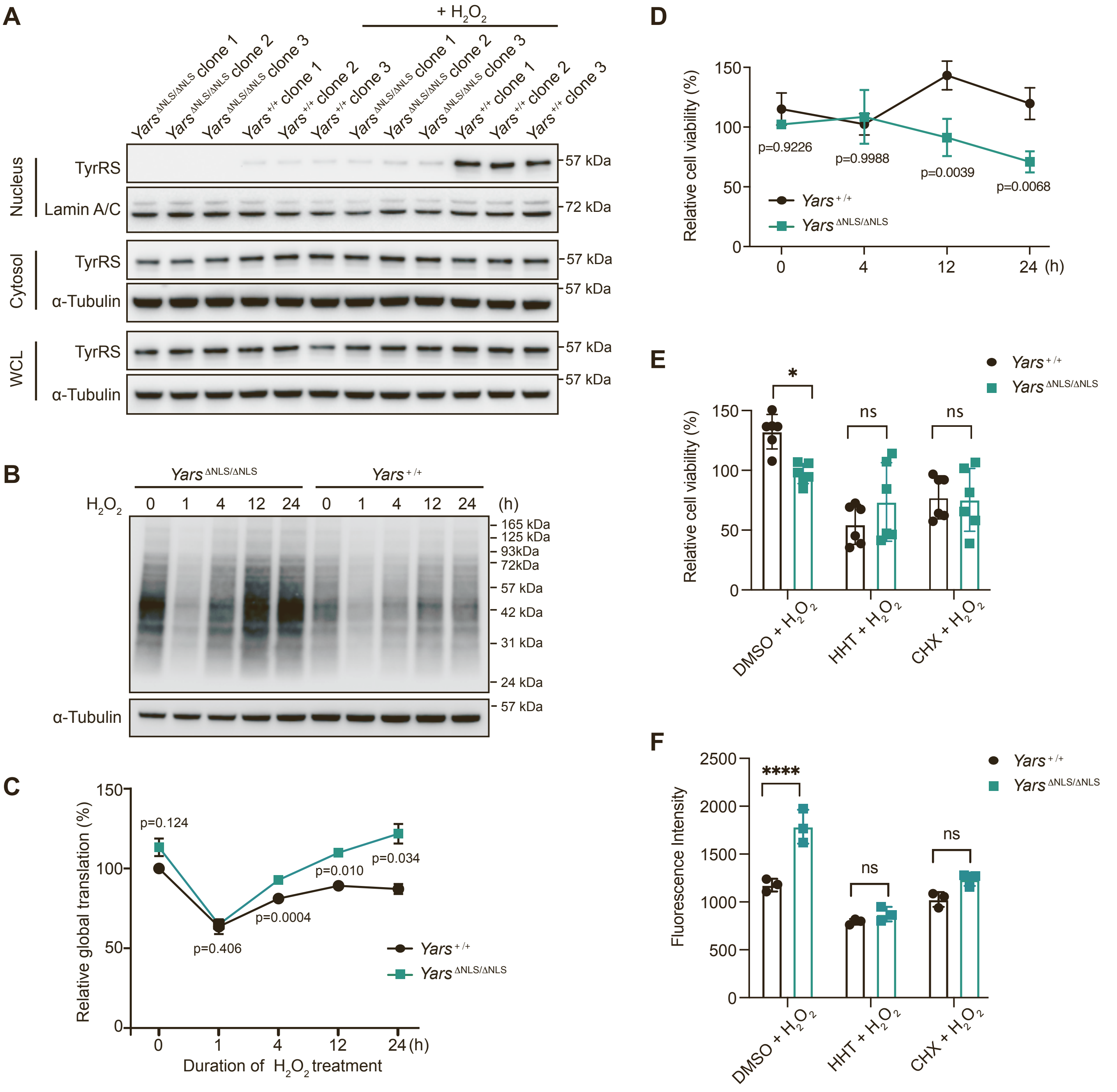

Figure S6: Nuclear TyrRS deficient mouse embryonic fibroblasts under oxidative stress exhibit an increased mRNA translation and are more prone to cell death, but protein synthesis inhibition decreases this cell death. Yars$^{+/+}$ MEFs derived from wild-type C57BL6/J. Yars$^{\Delta NLS/\Delta NLS}$ MEFs derived from nuclear TyrRS deficient C57BL6/J mice (^{242}KKKLKK^{247} to ^{242}NNKLNK^{247}).

A) Cell fractionation and western blot analysis of primary MEFs confirm disruption of nuclear import in Yars$^{\Delta NLS/\Delta NLS}$ cells compared to Yars$^{+/+}$ even upon oxidative stress exposure. Three independent mouse embryos (clones 1-3) were used for each genotype. Cells treated with H_2O_2 for 12 hours followed by fractionation. Lamin A/C: nuclear marker; α-Tubulin: cytoplasmic marker; WCL: Whole Cell Lysate.

B) SUnSET analysis of wild-type and nuclear TyrRS deficient MEFs. Cells treated with 200 μM H_2O_2 for the indicated time. α-Tubulin: loading control

C) Quantification of protein synthesis activity measured by SUnSET. Signal intensities for each treatment were normalized to α-Tubulin and Yars$^{+/+}$ at timepoint 0 hour was set to 100%. n=3, biological replicates, Student's t-test.

D) Nuclear TyrRS deficient MEFs have increased cell death from H_2O_2 (200 μM) treatment. Relative cell survival of untreated cells set to 100%. n=3, biological replicates, Student's t-test.

E) Translation inhibition by HHT (50 nM) or CHX (50 ng/mL) reduced cell death in nuclear TyrRS deficient MEFs. Cells treated with inhibitors for 24 hours followed by 24 hours of H_2O_2 treatment. Relative cell survival of untreated cells set to 100%. n=3, Student's t-test, $p<0.05$, ns: not significant.

F) Nuclear TyrRS lessened ROS production after 24 hours, and this effect is reduced with translation inhibitors homoharringtonine (HHT, 50 nM) and cycloheximide (CHX, 50 ng/mL) treatment 24 hours pre-oxidative stress. ROS production measured by CM-H2DCFDA and DMSO only treated Yars$^{+/+}$ cells set to 100%. n=3, Student's t-test, $p<0.0001$, ns: not significant.

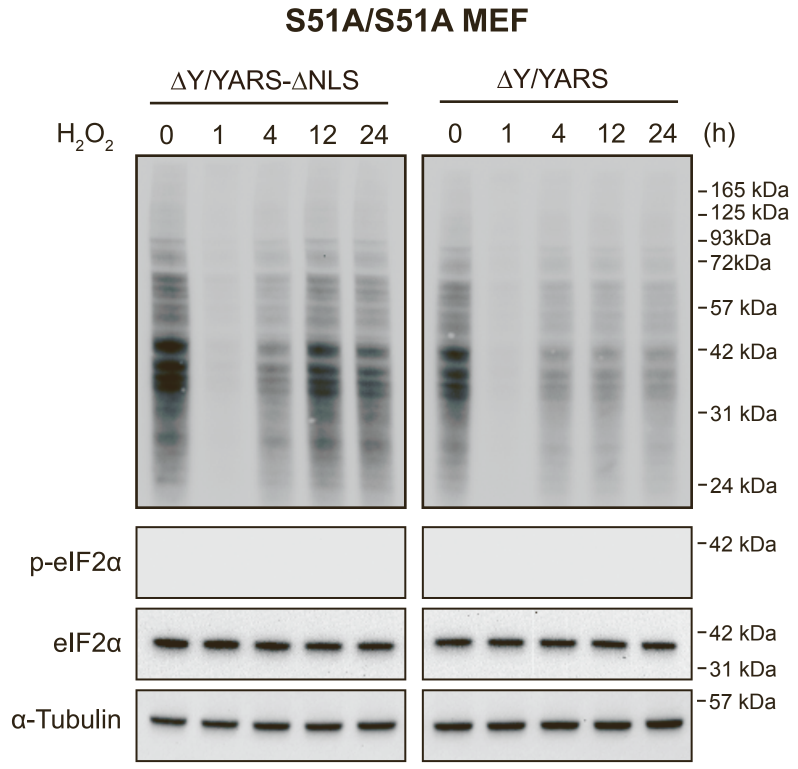

Figure S7: SUnSET analysis to detect protein synthesis activity in eIF2α phosphorylation deficient (eIF2α^{S51A}) mouse fibroblasts with or without nuclear TyrRS deficiency. Cells were treated with H_2O_2 for the indicated time. Total and phosphorylated eIF2α were analyzed by western blot using whole cell lysate to confirm eIF2α phosphorylation deficiency. ΔY/YARS-ΔNLS: eIF2α^{S51A} cells with a knock down of endogenous TyrRS and expression of TyrRS with a mutated NLS (^{242}KKKLKK^{247} to ^{242}NNKLNK^{247}). ΔY/YARS: eIF2α^{S51A} cells with a knock down of endogenous TyrRS and ectopic expression of wild-type TyrRS.

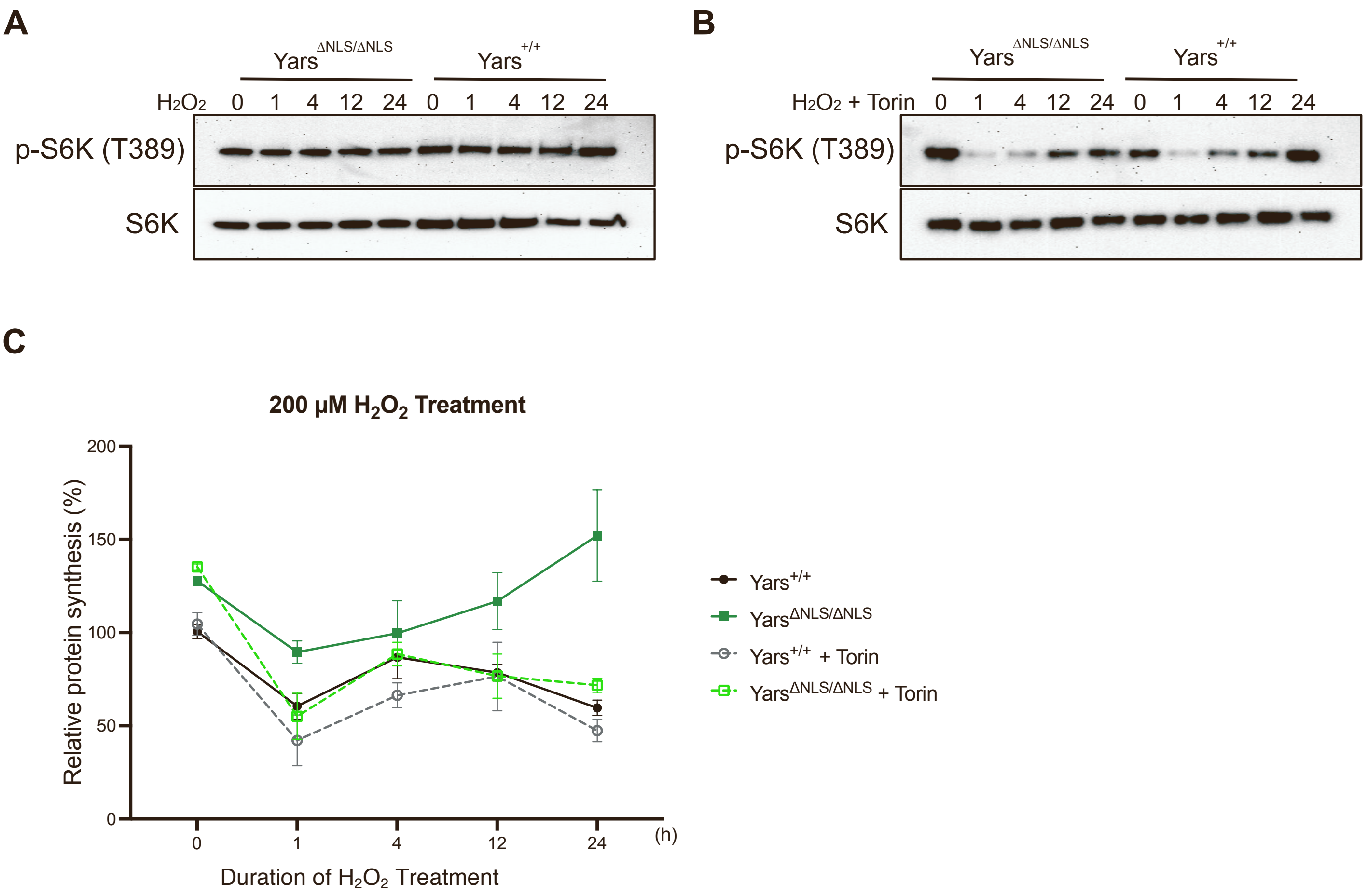

Figure S8: Investigations on mTOR activity and its impact on global translation in nuclear TyrRS deficient mouse embryonic fibroblasts.

A) No substantial change in p70-S6K (S6K) phosphorylation status between nuclear TyrRS deficient and control fibroblasts upon H_2O_2 treatment. p70-S6K antibody against the T389 phosphorylation site.

B) Both nuclear TyrRS deficient and control fibroblasts show a decreased phosphorylation of S6K upon simultaneous treatment of H_2O_2 and Torin (31.25 nM) for at least 12 hours.

C) Western blot quantification of protein synthesis activity measured by SUnSET of nuclear TyrRS deficient and control fibroblasts following Torin treatment. All cells treated with H_2O_2 for the indicated times. Signal intensities for each treatment were normalized to α-Tubulin and further normalized to $Yars^{+/+}$ at timepoint 0 hour (set to 100%).

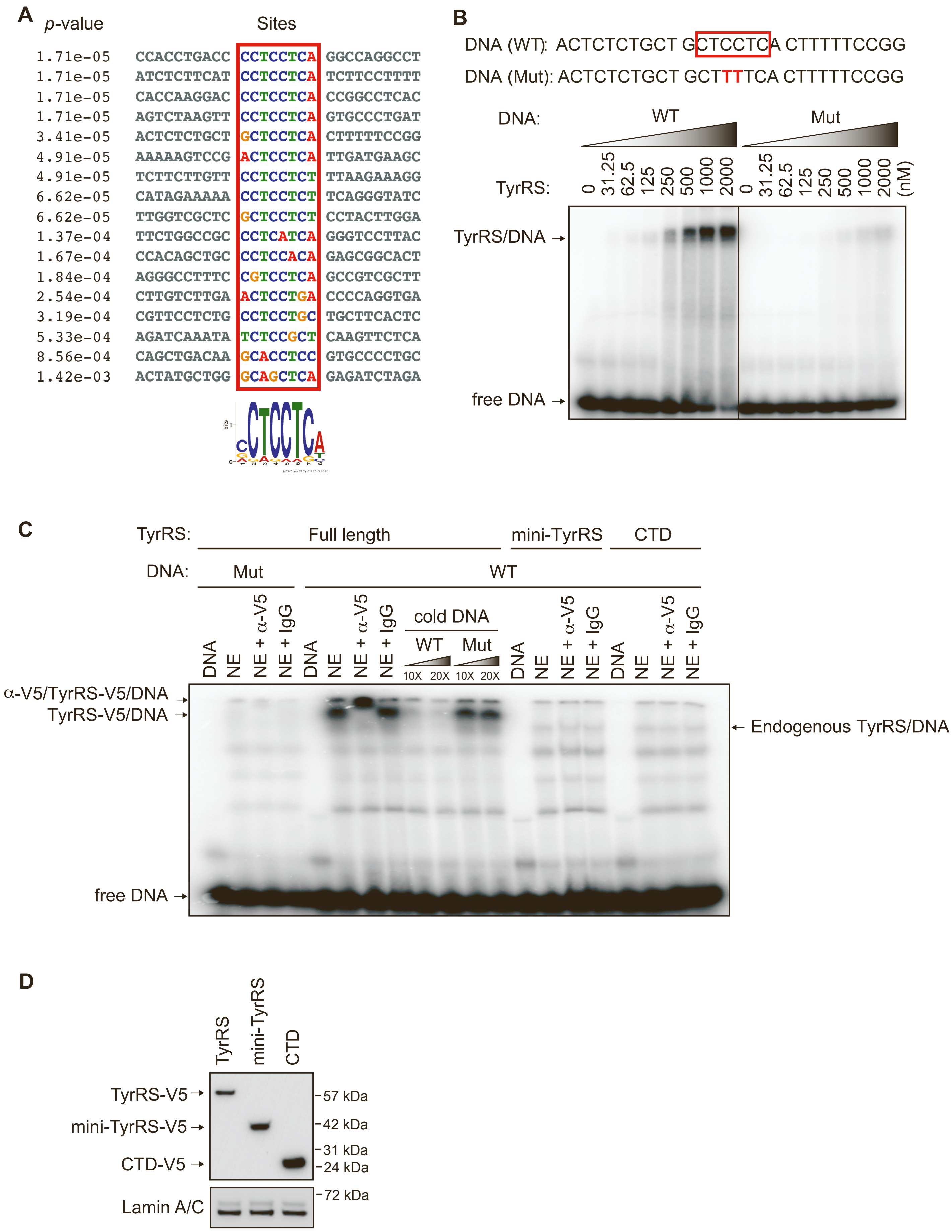

Supplementary Figure 9

Figure S9: Analysis of TyrRS DNA binding sites.

(A) Motif analysis of TyrRS binding sites. The DNA fragments identified by ChIP-seq were analyzed with MEME software.

(B,C) DNA binding ability and specificity of TyrRS *in vitro* (B) and in the context of cell nuclear extraction (NE) (C) as detected by EMSA. B) TyrRS purified from mammalian cells binds to the wild-type probe with a CTCCTC motif but not to the mutated probe. C) The nuclear fraction of cells expressing TyrRS-V5, but not mini-TyrRS-V5 or CTD-V5, binds to a probe with the CTCCTC motif. Competition between cold and hot probe with CTCCTC motif confirms binding specificity. Super shift with α-V5 antibody confirms the formation of TyrRS-V5/DNA complex. NE were prepared from HEK293 cells with transgene overexpression for 24 hours.

(D) Western blot analysis to show the expression level of the V5-tagged TyrRS, mini-TyrRS, and CTD in HEK293 cells overexpressing each construct. Because of the small size, CTD can enter the nucleus without an NLS. Lamin A/C: loading control.

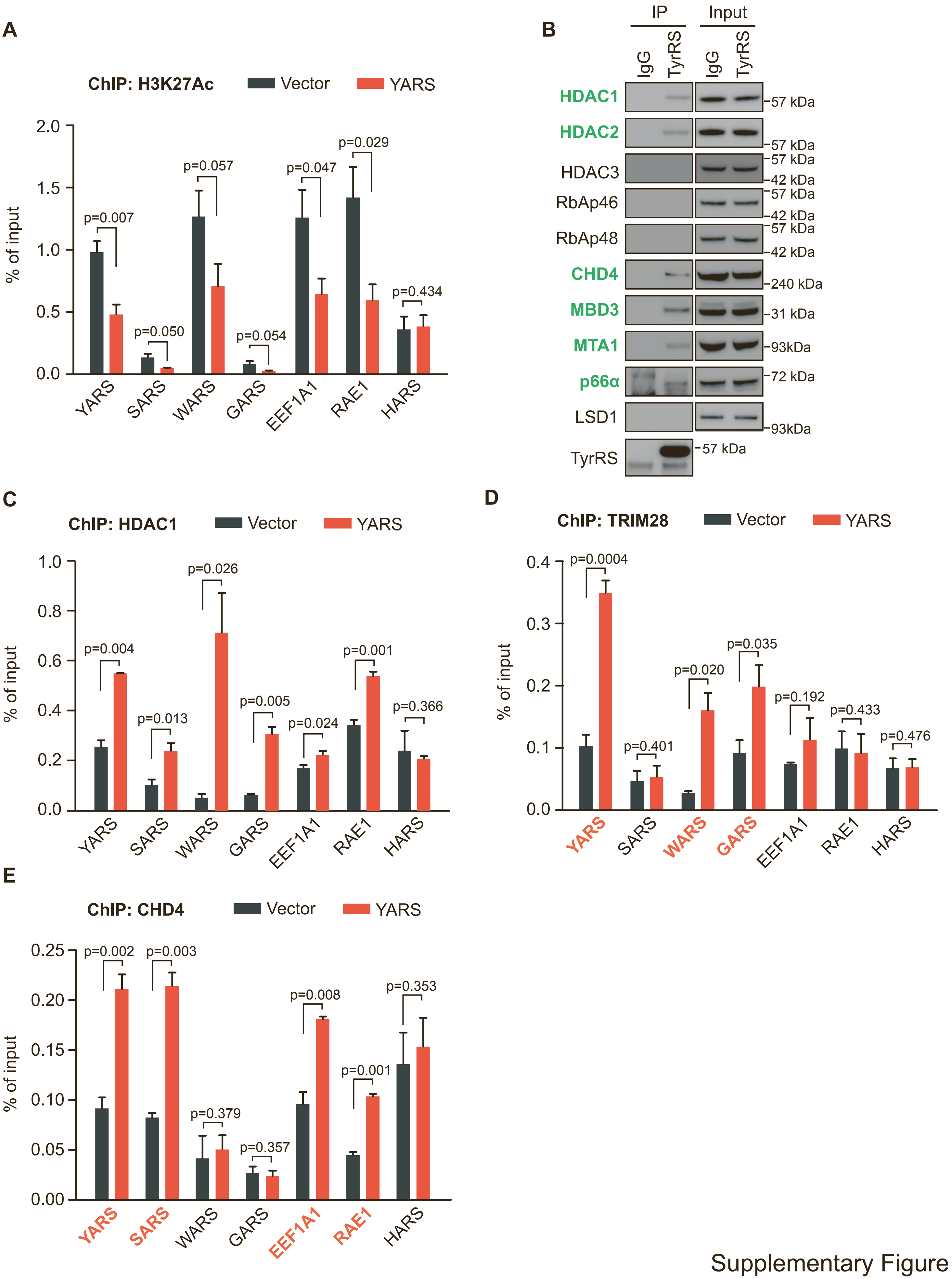

Supplementary Figure 10

Figure S10. TyrRS recruits transcriptional co-repressor TRIM28/HDAC1 or NuRD complex to epigenetically repress expression of its target genes. All ChIP-qPCR assays were done using HEK293 cells with vector or TyrRS overexpression for 24 hours. Vector: control, YARS: TyrRS overexpression

A) TyrRS represses histone H3 acetylation on its target sites. The enrichment of histone H3 acetylation was determined by chromatin IP using α-H3K27Ac antibody followed by qPCR. n=3, biological replicates, Student's t-test.

B) TyrRS interacts with HDAC1 and other factors in the NuRD complex. Co-immunoprecipitation using α-TyrRS antibody followed by western blot analysis to detect proteins involved in the NuRD complex.

C) TyrRS recruits HDAC1 to its target sites on translation-related genes in TyrRS overexpressing cells. Enrichment of HDAC1 was detected by chromatin IP using α-HDAC1 antibody followed by qPCR. n=3, biological replicates, Student's t-test.

D) Recruitment of TRIM28 at multiple TyrRS target sites (YARS1, WARS1, and GARS1) in TyrRS overexpressing HEK293 cells. Enrichment of TRIM28 was detected by chromatin IP using α-TRIM28 antibody followed by qPCR. n=3, biological replicates, Student's t-test.

E) Increased occupancy of CHD4, a component of the NuRD complex factor, at multiple TyrRS target sites (YARS1, SARS1, EEF1A1, and RAE1) in TyrRS overexpressing HEK293 cells. Enrichment of CHD4 was detected by chromatin IP using α-CHD4 antibody followed by qPCR. n=3, biological replicates, Student's t-test.

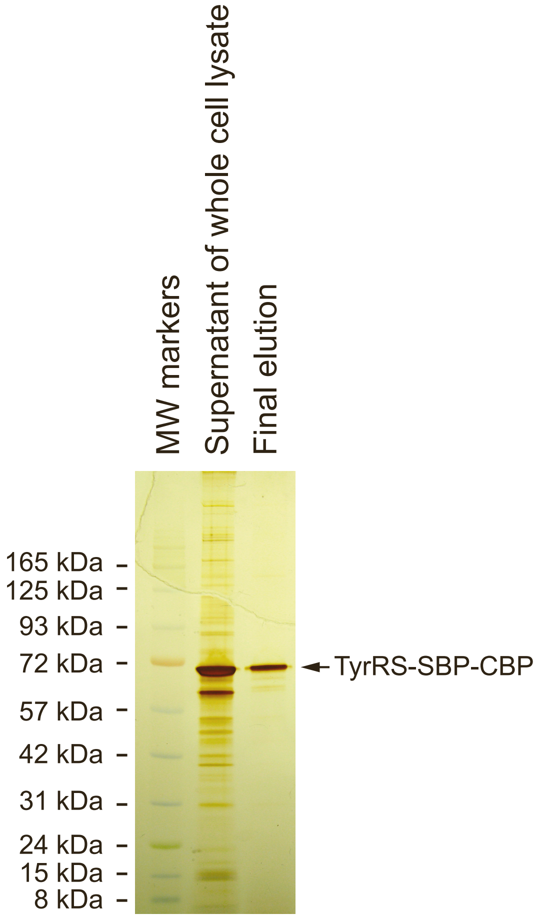

Figure S11: Silver staining showing the stringent Tandem Affinity Purification for isolating TyrRS and its interactome for mass spectrometry (TAP-MS) analysis. TyrRS-SBP-CBP: the fusion protein of TyrRS with a streptavidin binding peptide (SBP) and a calmodulin binding peptide (CBP).

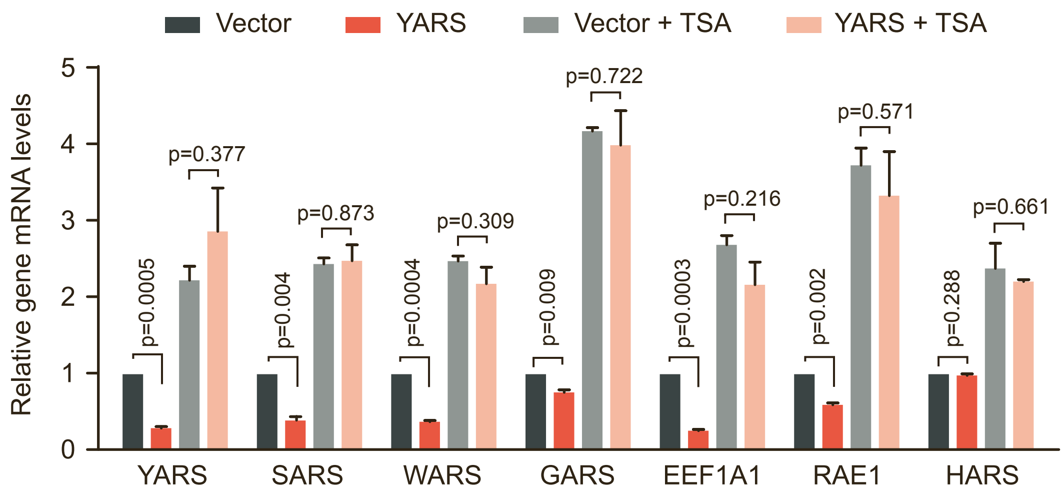

Figure S12: HDAC inhibitor Trichostatin A (TSA) treatment blocks the inhibitory effect of overexpressed TyrRS on its target gene transcription. Transcription of target genes were measured by RT-PCR using HEK293 cells with transgene overexpression for 24 hours, followed by DMSO or TSA treatment for another 24 hours. n=3, biological replicates, Student's t-test. Vector: control, YARS: TyrRS overexpression.

A

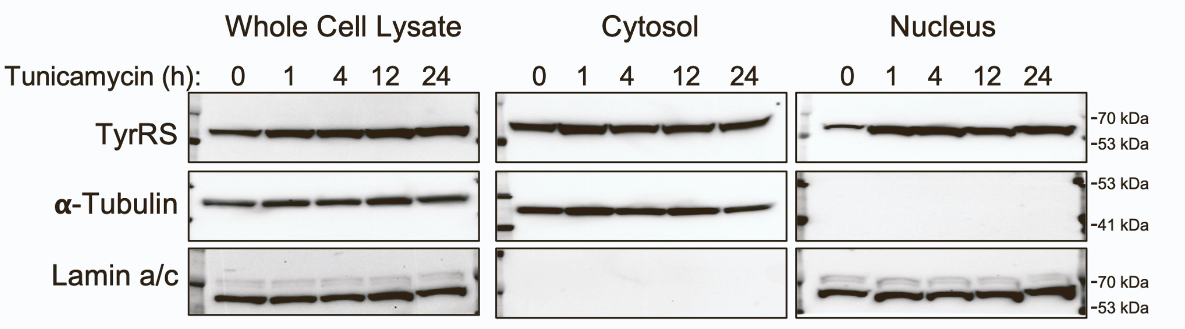

B

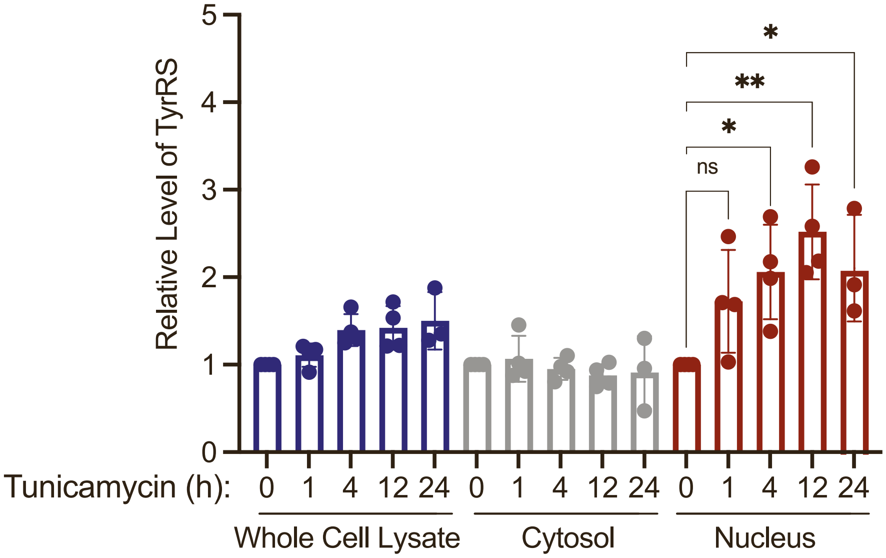

Figure S13: Tunicamycin treatment, which induces ER stress, induces TyrRS nuclear translocation in HEK-293T cells.

A) Representative western blot images after cell fractionation of tunicamycin (5 µg/mL) treated HEK-293T cells. Lamin A/C: nuclear marker; α-Tubulin: cytoplasmic marker.

B) Quantification of the western blots with TyrRS signals normalized to α-Tubulin or Lamin A/C, and that in untreated (timepoint zero) cells. $^{**}p<0.01$, $^{*}p<0.05$, n.s. not significant, n=3-4, biological replicates; one-way ANOVA test with Dunn's multiple comparison test.

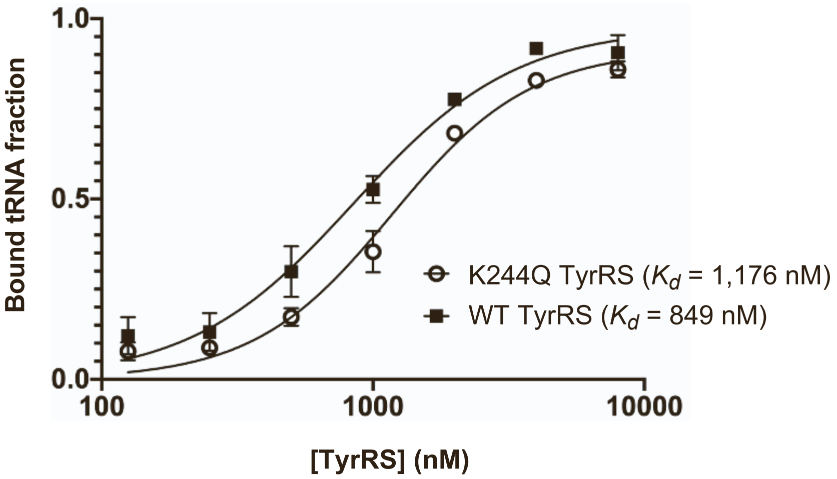

Figure S14: *In vitro* filter binding assay to determine the dissociation constant of WT and K244Q TyrRS for binding to its cognate tRNA transcript. n=3.

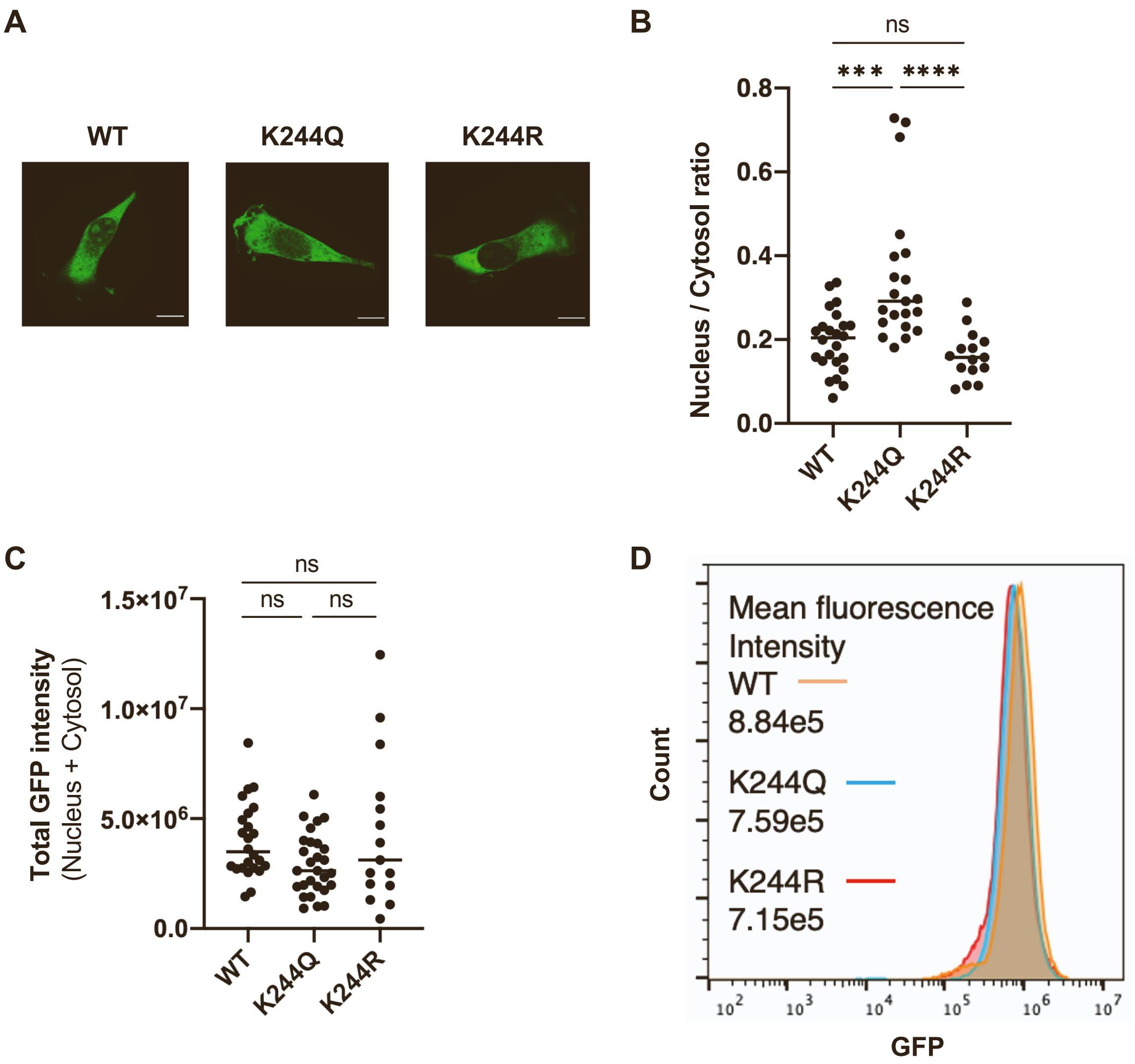

Figure S15: Quantitative analysis of TyrRS-GFP with acetylation (K244Q) or deacetylation (K244R) mimic mutations in B16-F10 cells, suggesting that acetylation enhances TyrRS nuclear localization.

A) Representative images of B16-F10 cells with WT, K244Q or K244R TyrRS-GFP expression. Scale bars = 10 μm.

B) Quantitative analysis of WT, K244Q or K244R TyrRS-GFP nuclear distribution as indicated by the ratio of the GFP intensity in the nucleus and cytosol. * * * $p < 0.001$, * * * * $p < 0.0001$, ns, not significant, by Kruskal-Wallis test with Dunn's multiple comparisons test.

C) The total (cytosol + nucleus) GFP intensity of the cells analyzed in (B) are comparable among the 3 cell lines. ns, not significant, determined by Kruskal-Wallis test with Dunn's multiple comparisons test.

D) The histogram of GFP intensity analyzed by flow cytometry to indicate expression level of the TyrRS-GFP proteins.
